# Serious adverse drug reactions at two children’s hospitals in South Africa

**DOI:** 10.1186/s12887-019-1892-x

**Published:** 2020-01-04

**Authors:** Johannes P. Mouton, Melony C. Fortuin-de Smidt, Nicole Jobanputra, Ushma Mehta, Annemie Stewart, Reneé de Waal, Karl-Günter Technau, Andrew Argent, Max Kroon, Christiaan Scott, Karen Cohen

**Affiliations:** 1 Department of Medicine, Division of Clinical Pharmacology, University of Cape Town, Cape Town, South Africa; 2 Centre for Infectious Disease Epidemiology and Research, School of Public Health and Family Medicine, University of Cape Town, Cape Town, South Africa; 3 Department of Paediatrics and Child Health, Empilweni Services and Research Unit, Rahima Moosa Mother and Child Hospital, School of Clinical Medicine, Faculty of Health Sciences, University of Witwatersrand, Johannesburg, South Africa; 4 Department of Paediatrics and Child Health, University of Cape Town, Cape Town, South Africa; 5 Department of Paediatrics and Child Health, Division of Neonatology, University of Cape Town, Cape Town, South Africa

**Keywords:** Adverse drug reaction, Pharmacoepidemiology, Prevalence, Trigger tool, HIV

## Abstract

**Background:**

The high HIV prevalence in South Africa may potentially be shaping the local adverse drug reaction (ADR) burden. We aimed to describe the prevalence and characteristics of serious ADRs at admission, and during admission, to two South African children’s hospitals.

**Methods:**

We reviewed the folders of children admitted over sequential 30-day periods in 2015 to the medical wards and intensive care units of each hospital. We identified potential ADRs using a trigger tool developed for this study. A multidisciplinary team assessed ADR causality, type, seriousness, and preventability through consensus discussion. We used multivariate logistic regression to explore associations with serious ADRs.

**Results:**

Among 1050 patients (median age 11 months, 56% male, 2.8% HIV-infected) with 1106 admissions we found 40 serious ADRs (3.8 per 100 drug-exposed admissions), including 9/40 (23%) preventable serious ADRs, and 8/40 (20%) fatal or near-fatal serious ADRs. Antibacterials, corticosteroids, psycholeptics, immunosuppressants, and antivirals were the most commonly implicated drug classes. Preterm neonates and children in middle childhood (6 to 11 years) were at increased risk of serious ADRs compared to infants (under 1 year) and term neonates: adjusted odds ratio (aOR) 5.97 (95% confidence interval 1.30 to 27.3) and aOR 3.63 (1.24 to 10.6) respectively. Other risk factors for serious ADRs were HIV infection (aOR 3.87 (1.14 to 13.2) versus HIV-negative) and increasing drug count (aOR 1.08 (1.04 to 1.12) per additional drug).

**Conclusions:**

Serious ADR prevalence in our survey was similar to the prevalence found elsewhere. In our setting, serious ADRs were associated with HIV-infection and the antiviral drug class was one of the most commonly implicated. Similar to other sub-Saharan African studies, a large proportion of serious ADRs were fatal or near-fatal. Many serious ADRs were preventable.

## Background

Patient safety is of central importance to all fields of medicine. However, in paediatric medicine, clinicians are faced with the reality that pre-registration drug safety data are often very limited, and that extrapolation from adult drug safety data may be inappropriate in view of children’s physiological development. As such, post-marketing surveillance is critical in the ongoing safety appraisal of drugs given to children [1].

South Africa was home to an estimated 280,000 children living with HIV in 2017, of which an estimated 58% were on antiretroviral treatment (ART) [2]. Despite this high prevalence of HIV, and despite ART’s notorious potential to cause adverse drug reactions (ADRs) and drug-drug interactions, drug safety is a relatively understudied field in South Africa. Local paediatric drug safety data could potentially contribute to clinical decision-making and health programme policy-making.

Our main aim with this survey was to describe the prevalence of serious ADRs in two paediatric hospitals in South Africa, including the prevalence of serious ADRs at the time of admission and the prevalence of serious ADRs occurring during the admission. Further aims were to describe serious ADR manifestations and the drugs implicated in serious ADRs, to describe the preventability of serious ADRs, to describe the prevalence and manifestations of non-serious ADRs in this setting, and to explore the influence of HIV on ADRs occurring in this patient population.

## Methods

### Design and setting

We conducted this observational study through folder review of data documented during routine clinical care of patients at two hospitals in South Africa: Red Cross War Memorial Children’s Hospital (RCWMCH), situated in Cape Town, Western Cape province, and Rahima Moosa Mother and Child Hospital (RMMCH), situated in Johannesburg, Gauteng province. We selected these sites based on existing research collaborations. Both sites have paediatric ART clinics.

At RCWMCH, we surveyed all patients admitted electively and non-electively over a 30-day period in April and May 2015 to the general and specialist medical wards, to high-care beds in the medical wards, to the combined paediatric intensive care unit, and to the general section (but not the rehydration section) of the short-stay ward. We excluded admissions to surgical or oncology wards if the entire stay was spent in the surgical and oncology wards, but included those portions of surgical and oncology patients’ admissions spent in the paediatric intensive care unit. The hospital provides limited neonatal services. Admission trends to RCWMCH have previously been described [3]. At RMMCH, we surveyed all patients admitted non-electively over a 30-day period in June and July 2015 to the medical wards and to the combined intensive care / high-care unit. Although RMMCH provides neonatology services, we only surveyed those neonates admitted to the intensive care / high-care unit, and not those admitted to the postnatal wards. Elective admissions to RMMCH, which were excluded from our survey, consisted mostly of children admitted as day patients for minor surgical procedures.

### Sample size considerations

We calculated that a sample of 514 patients would detect a prevalence of serious ADRs present at admission of 2.9% (based on a previous systematic review [4]) with a 95% confidence interval (CI) of 1.6 to 4.8%, using the exact CI method of Clopper-Pearson [5]. Based on historic admission trends at the sites and specific wards included in the survey, we were confident that we would survey at least 514 admissions during the planned study duration.

### Study processes

We consulted hospital administrative records every weekday during the 30-day survey period to identify all new admissions. The survey team (a general practitioner and a pharmacist) reviewed each patient’s clinical notes, medication prescription charts, and laboratory results as soon as possible after admission and approximately every second day thereafter until the patient’s discharge or death, or until study closure, seven days after the end of the 30-day survey entry period. At the time of the patient’s discharge, the survey team specifically reviewed the discharge summary prepared by the clinical team to verify and augment data already collected. For patients admitted to the RCWMCH short-stay ward, we conducted folder reviews retrospectively approximately five months following the admission, and not prospectively as described above, as piloting showed it to be too resource-intensive to survey admissions prospectively in this ward.

We abstracted demographic and clinical data (including medication histories) from all patients using electronic case report forms. The survey team identified *potential* ADRs during the folder review with the help of a trigger tool (Table 1, development described below). For cases flagged by the trigger tool, we abstracted a more detailed dataset, including relevant laboratory results, and details on the management and outcome of the potential ADR. In a second stage of ADR identification and assessment, a multidisciplinary team (the survey team, together with a paediatrician, a paediatrician highly experienced in neonatology, a clinical pharmacologist, and a clinical pharmacist) discussed flagged cases’ abstracted data to reach consensus on causality (thereby determining whether the potential ADR was actually an ADR), ADR type, seriousness, and preventability, using definitions described below.

**Table 1:** Trigger list used to assist in identifying potential adverse drug reactions

Category	Trigger
Use of these drugs (antidotes) suggests a potential ADR occurred	Naloxone
	Methadone
	Flumazenil
	Digoxin immune fab (“Digibind”)
	Protamine sulphate
	Activated charcoal
	Biperiden / promethazine / diazepam
	Sodium polystyrene (“Kayexalate”)
	Insulin with glucose
	Calcium gluconate
	Dextrose 10%
	Adrenaline (epinephrine)
	Systemic corticosteroid
	Diphenhydramine, prochlorperazine, promethazine, or any new antihistamine
	Antiemetics
	Oral vancomycin
	Granulocyte colony-stimulating factor
Supratherapeutic drug concentration suggests a potential ADR occurred	Digoxin > 1.5 nmol/L
	Theophylline > 110 μmol/L
	Lidocaine > 5 μg/L
	Phenytoin > 80 μmol/L
	Carbamazepine > 51 μmol/L
	Phenobarbital > 172 μmol/L
	Valproic acid > 700 μmol/L
	Gentamicin or tobramycin peak > 10 mg/L or 24 h trough > 2 mg/L
	Amikacin peak > 30 mg/L or 24 h trough > 2 mg/L
	Vancomycin trough > 25 mg/L or > 30 mg/L during continuous infusion
	Any other drug concentration reported as supratherapeutic
	Paracetamol concentration done (regardless of result)
Laboratory result (other than TDM) suggests a potential ADR occurred	Partial thromboplastin time (PTT) > 100 s
	International normalised ratio (INR) > 5
	Anti-factor Xa ≥ 1.5 IU/mL
	Platelet count < 50 × 10^9^/L
	White cell count < 3 × 10^9^/L
	Haemoglobin < 8 g/dL
	Pancytopaenia
	*Clostridium difficile* positive stool after exposure to antibiotics
	Creatinine rising to above-normal range
	Hyponatraemia or Hypernatraemia
	Potassium < 3.5 mmol/L, in the absence of diarrhoea
	Potassium > 5.5 mmol/L
	Alanine transaminase (ALT) > three times upper limit of normal (ULN = 40 IU/L) in the presence of negative viral hepatitis screening test results
	Bilirubin > two times upper limit of normal (ULN = 21 μmol/L); if in a neonate, the neonate should also be on drugs
	Serum glucose < 3 mmol/L, outside the perinatal period
	Hyperlactataemia on antiretroviral therapy
Clinical event suggests a potential ADR occurred	Angioedema or lip swelling
	Rash or ulceration
	Mucositis or mucosal ulceration
	Pruritus
	Sudden onset wheezing
	Jaundice, new onset
	Dystonia, ataxia, torticollis, dyskinesia
	Retinopathy in premature infant on oxygen
	Hearing disturbance or hearing loss
	Seizure(s)
	Oversedation, lethargy, falls
	Decreased level of consciousness or pressure sores
	Delirium
	Fracture or osteoporosis
	Upper gastrointestinal bleed
	Arrhythmia, new
	Hypertension
	Nausea reported by parents / documented in file
	Constipation
	Biopsy of bone marrow, kidney, or liver
	Withdrawal symptoms
	Any other event suspected to be drug-related by doctor or nurse
	Unexplained medication stop
	Readmission to acute care unit within 14 days of discharge
	Readmission to intensive care unit within 48 h of transfer or discharge
	Require resuscitation in ward
	Death

### Definitions, classifications, and taxonomies

We defined ADRs according to the 2005 Aronson and Ferner definition [6]. We performed causality assessment according to the World Health Organization (WHO)-Uppsala Monitoring Centre system for standardised case causality assessment [7]. Those potential ADRs where drug causation was assessed as certain, probable, or possible were counted as ADRs. We specifically did not consider the following scenarios to be ADRs: intentional drug overdose, poisoning by or ingestion of non-medicinal products, poisoning by herbal or traditional remedies, therapeutic failures, complications associated with poor adherence, and medication errors which were not associated with any harm. We codified ADRs to ‘preferred terms’ in version 17.1 of the Medical Dictionary for Regulatory Activities (MedDRA®, MedDRA Maintenance and Support Services Organization, McLean, VA, USA). We classified ADRs as preventable if at least one of the Schumock and Thornton preventability questions [8] were answered affirmatively by the multidisciplinary team. We classified ADRs in accordance with the Rawlins and Thompson classification as type A or type B [9]. We used this classification to decide the level at which we conducted causality and preventability assessment in the event that multiple drugs were implicated in the ADR, as described in a previous paper by our group [10]. If multiple drug suspects were implicated in a type A ADR, we assessed causality and preventability based on the combined action of all the drug suspects. If multiple drug suspects were implicated in a type B ADR, we assessed causality and preventability for each drug suspect–ADR pair separately. We categorised the seriousness of ADRs as per Temple [11], as causing: (i) increased monitoring but no harm; (ii) temporary harm, requiring treatment intervention; (iii) initial / prolonged hospitalization; (iv) permanent harm; (v) near-death; or (vi) death. We defined serious ADRs as those ADRs resulting in hospital admission, or prolonging hospital admission, or causing permanent harm, near-death, or death.

We recorded drugs by generic names only and codified these according to the World Health Organization’s Anatomical Therapeutic Chemical Classification System (ATC) [12]. We defined the total drug count as the number of unique ATC codes to which the patient was exposed over the 30-day period before the admission and during their observed hospital stay. We similarly calculated a background drug count (over the 30-day period before the admission) and an in-hospital drug count for each admission. When determining drug counts, we excluded drugs applied topically to the skin, eyes, ears, nose, throat, or mouth.

We categorised age according to National Institute of Child Health and Human Development Pediatric Terminology as follows: preterm neonate, born before 37 completed weeks’ gestation; term neonate, from birth to 27 days; infancy, 28 days to 12 months; toddler, 13 months to 24 months; early childhood, 25 months to 5 years; middle childhood, 6 years to 11 years; early adolescence, 12 years to 18 years [13]. We used the admission weight to calculate a weight-for-age z-score using standards developed in the WHO Multicentre Growth Reference Study [14] for infants, toddlers, and children in early childhood years. We abstracted HIV status as infected, negative, or unknown, and perinatal HIV exposure status in children < 18 months old as exposed, unexposed, or unknown. We combined HIV status and perinatal HIV exposure into a single stratified variable as follows: [1] HIV-infected; [2] HIV-negative, consisting of children < 18 months who were not perinatally exposed, children ≥18 months who were serologically negative, and children ≥18 months in whom HIV testing was not clinically indicated; and [3] an indeterminate group of children < 18 months, who were or may have been perinatally exposed to HIV but whose HIV infection status was not yet confirmed.

### Trigger tool development

We conducted a literature review to identify previous studies that used triggers to detect potential ADRs or harm in children, or potential ADRs in adults. We combined all the drug-related triggers from these various tools. A multidisciplinary panel of seven experts (a clinical pharmacist, a clinical pharmacologist, two paediatricians, a paediatric HIV clinician, and two research medical officers) then decided on the inclusion of each trigger in a two-round modified Delphi method. In round 1, each expert independently rated the inclusion of the trigger on a 5-point Likert scale (5 = strongly agree that the trigger should be included on the trigger tool; 1 = strongly disagree that the trigger should be included on the trigger tool). We calculated the median score, first (Q1), and third (Q3) quartiles for each trigger. We defined agreement to include each potential trigger as a median score ≥ 4 and Q1 ≥ 4, and agreement to exclude each potential trigger as a median score ≤ 2 and Q3 ≤ 2. Additionally, experts were asked to add potential triggers to the list during round 1. In round 2, experts met to discuss triggers without agreement to include or exclude, as well as all the potential triggers added during round 1. Experts then re-scored, or in the case of newly added triggers scored, these triggers independently on the same scale. We again defined agreement to include each potential trigger as a median score ≥ 4 and Q1 ≥ 4. Triggers with agreement to include in the first or second round were included in the final trigger list.

### Data management and statistical analysis

We entered data into a purpose-built Access 2013 database (Microsoft Corporation, Redmond, WA). We analysed data using Stata 13.1 (Stata Corporation, College Station, TX), including the macro igrowup_restricted.ado (version 3.2.2, January 2011) [15] to calculate weight-for-age z-scores.

We summarised continuous variables by means and standard deviations, or by medians and interquartile ranges (IQR), depending on their distribution. We explored associations between binary and categorical variables through cross-tabulation and chi-square statistics, and we conducted between-group comparisons of continuous variables using the Wilcoxon rank-sum test or Student’s t test, depending on the distribution. A *P* value of < 0.05 was taken to indicate statistically significant difference.

We conducted multivariate analysis of associations with serious ADR by constructing a logistic regression model. We limited this model to children documented to have been exposed to at least one drug before and / or during their admission, and to first admissions only in the event of multiple admissions per patient. Variables which were selected a priori for inclusion in the model as predictor variables were age category, sex, hospital site, the summary HIV infection / exposure category described above, and total drug count. In the subgroup of patients for whom weight-for-age z-scores could be calculated, we conducted an explorative analysis adding this variable to the model.

### Ethical issues

Our study received ethical approval from the Human Research Ethics Committees of the University of Cape Town (approval number 576/2011) and the University of the Witwatersrand (clearance certificate number M140707). We received permission to conduct the research from both hospitals. We did not request individual patients’ or caregivers’ consent, as this study was a non-interventional review of medical records, and this was approved by the ethics committees. We shared anonymised study findings with the South African National Adverse Drug Event Monitoring Centre, which collects spontaneous reports on behalf of the national medicines regulator. Partial, provisional results were shared at the 32nd International Conference on Pharmacoepidemiology & Therapeutic Risk Management and were published as an abstract [16].

## Results

### Trigger tool development

Our literature search yielded 31 articles. We compiled a list of 110 triggers from 16 included studies (see Additional file 1: Table S1). In round 1 of the modified Delphi method, we agreed to include 37 triggers and agreed to exclude one. In round 2, 74 triggers (72 from round 1 and two newly proposed triggers) were discussed. We agreed to include a further 35; the final trigger list consisted of 72 triggers (Table 1). Seventeen triggers refer to drugs used as antidotes (e.g. naloxone) or in the management of adverse events, 12 describe laboratory evidence of high drug concentrations, 16 refer to other abnormal laboratory values, and 27 relate to clinical events suggesting adverse drug events (e.g. unexplained medication stop.)

### Sample description

There were 1050 patients and 1106 admissions (range one to four admissions per patient). Patient characteristics are described in Table 2, and admission characteristics in Table 3.

**Table 2:** Patient characteristics (*n* = 1050) at first admission to two children’s hospitals, South Africa, 2015

	All patients (*n* = 1050)	Patients with ADR(s) (any seriousness) (*n* = 119)	Patients without ADR(s) (*n* = 931)	*P*-value
Age				
Median (IQR) months	11 (2 to 41)	20 (5 to 68)	10 (2 to 37)	*P* = 0.0010^e^
Age categories^a^				
Preterm neonates	32 (3.1%)^b, c^	6 (5.0%)	26 (2.8%)	*P* = 0.002^f^
Term neonates	96 (9.1%)^c^	2 (1.7%)	94 (10%)	
Infants	421 (40%)	42 (35%)	379 (41%)	
Toddlers	139 (13%)	13 (11%)	126 (14%)	
Early childhood	198 (19%)	30 (25%)	168 (18%)	
Middle childhood	123 (12%)	18 (15%)	105 (11%)	
Early adolescence	41 (3.9%)	8 (6.7%)	33 (3.5%)	
Weight-for-age z-score mean (SD)				
Infants	−1.3 (2.0) (*n* = 418)	−2.4 (2.2) (*n* = 42)	−1.2 (2.0) (*n* = 376)	*P* = 0.0002^g^
Toddlers	−0.45 (1.5) (*n* = 137)	−0.78 (1.2) (*n* = 13)	−0.41 (1.5) (*n* = 124)	*P* = 0.3870^g^
Early childhood	−0.54 (1.6) (*n* = 165)	−0.54 (1.7) (*n* = 24)	− 0.54 (1.5) (*n* = 141)	*P* = 0.9969^g^
Sex				
Male	590 (56%)	69 (58%)	521 (56%)	*P* = 0.676^h^
Female	460 (44%)	50 (42%)	410 (44%)	
HIV infection / exposure status				
HIV negative	887 (84%)	91 (76%)	796 (86%)	*P* = 0.003^f^
HIV indeterminate	134 (13%)	19 (16%)	115 (12%)	
HIV infected	29 (2.8%)^d^	9 (7.6%)	20 (2.1%)	
Number of admissions				
One	1001 (95%)	109 (92%)	892 (96%)	*P* = 0.134^f^
Two	43 (4.1%)	9 (7.6%)	34 (3.7%)	
Three	5 (0.48%)	1 (0.84%)	4 (0.43%)	
Four	1 (0.10%)	0 (0%)	1 (0.11%)	

^a^Preterm neonate, born before 37 completed weeks’ gestation; term neonate, from birth to 27 days; infancy, 28 days to 12 months; toddler, 13 months to 24 months; early childhood, 25 months to 5 years; middle childhood, 6 years to 11 years; early adolescence, 12 years to 18 years

^b^Preterm neonates included 16/32 (50%) moderate to late preterm (32 to < 37 weeks’ gestation), 12/32 (38%) very preterm (28 to < 32 weeks’ gestation), and 4/32 (13%) extremely preterm (< 28 weeks’ gestation) neonates

^c^Among all neonates (*n* = 128): 93/128 (73%) normal or high birth weight (≥2500 g), 18/128 (14%) low birth weight (1500 to 2499 g), 11/128 (8.6%) very low birth weight (1000 to 1499 g), and 6/128 (4.7%) extremely low birth weight (≤999 g)

^d^Of whom 5/29 (17%) not yet on ART, and 24/29 (83%) on ART before and during admission. ART regimens were: 15/24 (63%) on abacavir (ABC) + lamivudine (3TC) + ritonavir-boosted lopinavir (LPV/r), 3/24 (13%) on ABC + 3TC + efavirenz (EFV), 2/24 (8.3%) on zidovudine (AZT) + 3TC + LPV/r, 1/24 (4.2%) on stavudine (D4T) + 3TC + LPV/r, 1/24 (4.2%) on AZT + 3TC + nevirapine (NVP), 1/24 (4.2%) on AZT + 3TC + EFV, and 1/24 (4.2%) on an unknown ART regimen

^e^Wilcoxon rank-sum test

^f^Fisher’s exact test

^g^Student’s t test

^h^Chi-square test

*ADR* adverse drug reaction; *IQR* interquartile range; *SD* standard deviation

**Table 3:** Admission characteristics (*n* = 1106) at two children’s hospitals in South Africa, 2015

	All admissions (*n* = 1106)	Admissions with ADR(s) (any seriousness) (*n* = 120)	Admissions without ADR(s) (*n* = 986)	*P*-value
Hospital				
RCWMCH	886 (80%)	100 (83%)	786 (80%)	*P* = 0.349^c^
RMMCH	220 (20%)	20 (17%)	200 (20%)	
Type of admission				
Acute admission	998 (90%)	113 (94%)	885 (90%)	*P* = 0.124^c^
Elective admission	108 (9.8%)	7 (5.8%)	101 (10%)	
Admission ward				
Short-stay ward only	514 (46%)	28 (23%)	486 (49%)	*P* < 0.001^c^
Medical wards, not ICU^a^	459 (42%)	60 (50%)	399 (40%)	
Any time spent in ICU	133 (12%)	32 (27%)	101 (10%)	
Duration of stay observed (censored at study end) (*n* = 1105)^b^				
1 day	72 (6.5%)	2 (1.7%)	70 (7.1%)	*P* < 0.001^c^
2 to 3 days	541 (49%)	27 (23%)	514 (52%)	
4 to 6 days	229 (21%)	20 (17%)	209 (21%)	
≥ 7 days	263 (24%)	71 (59%)	192 (19%)	
Median (days)	3	8	3	*P* < 0.0001^e^
Interquartile range (days)	2 to 6	4 to 13	2 to 5	
Range (days)	1 to 34	1 to 34	1 to 34	
Total stay observed (patient-days)	5744	1198	4546	
Type of exit (*n* = 1105)^b^				
Discharged / transferred out	1038 (94%)	100 (83%)	938 (95%)	*P* < 0.001^d^
Died	13 (1.2%)	2 (1.7%)	11 (1.1%)	
Censored (remained in-hospital by end of observation period)	54 (4.9%)	18 (15%)	36 (3.7%)	
Drug exposure over 30-day period before admission				
No drug history documented in folder	226 (20%)	12 (10%)	214 (22%)	*P* < 0.001^c^
No drugs	130 (12%)	7 (5.8%)	123 (12%)	
Had ≥1 drug(s)	750 (68%)	101 (84%)	649 (66%)	
Median background drug count	2 (*n* = 880)	4 (*n* = 108)	2 (*n* = 772)	*P* < 0.0001^e^
Interquartile range	1 to 4	2 to 7	1 to 4	
Range	0 to 47	0 to 42	0 to 47	
Drug exposure during admission (*n* = 1105)^b^				
No drugs	69 (6.2%)	2 (1.7%)	67 (6.8%)	*P* = 0.028^c^
Had ≥1 drug(s)	1036 (94%)	118 (98%)	918 (93%)	
Median in-hospital drug count	4 (*n* = 1105)	11 (*n* = 120)	4 (*n* = 985)	*P* < 0.0001^e^
Interquartile range	3 to 9	7 to 19	2 to 7	
Range	0 to 46	0 to 46	0 to 36	
Median daily drug count	5 (*n* = 5744 patient-days)	9 (*n* = 1198 patient-days)	4 (*n* = 4546 patient-days)	*P* < 0.0001^e^
Interquartile range	3 to 8	5 to 13	2 to 7	
Range	0 to 29	0 to 29	0 to 23	
Total drug exposure (before and during admission)				
No drugs	49 (4.4%)	0 (0%)	49 (5.0%)	*P* = 0.012^c^
Had ≥1 drug(s)	1057 (96%)	120 (100%)	937 (95%)	
Median total drug count	6	12	5	*P* < 0.0001^e^
Interquartile range	3 to 10	8 to 21	3 to 9	
Range	0 to 52	2 to 47	0 to 52	

^a^May have moved from short-stay to medical ward; may have included a high-care stay during the admission

^b^Duration of stay, drug exposure during admission, and type of exit missing for one admission (an admission without an ADR)

^c^Chi-square test

^d^Fisher’s exact test

^e^Wilcoxon rank-sum test

*ADR* adverse drug reaction; *ICU* intensive care unit; *RCWMCH* Red Cross War Memorial Children’s Hospital; *RMMCH* Rahima Moosa Mother and Child Hospital

Respiratory infections were the most common reason for admission, forming one-third of admission diagnoses (see Additional file 1: Table S2). Beta-adrenergic inhalants, antipyretics, and penicillins and other beta-lactam antibiotics were the most common drugs to which children were exposed prior to their admission (see Additional file 1: Table S3), although one in five folders did not contain a pre-admission drug exposure history. The same drug classes, together with vitamin and mineral supplements, were also the most commonly used drugs during admissions (see Additional file 1: Table S4).

There were 29/1050 (2.8%) HIV-infected children: 24 were on ART before the admission, while five were newly diagnosed during the index admission and referred to start ART after discharge. Most (19/24, 79%) were on a regimen of ritonavir-boosted lopinavir and two nucleoside reverse transcriptase inhibitors (NRTIs), while 4/24 (17%) were on a regimen of a non-nucleoside reverse transcriptase inhibitor and two NRTIs, and 1/24 (4.2%) on an unknown regimen. The NRTIs in use were lamivudine in all 23 children, abacavir in 18, zidovudine in 4, and stavudine in 1. Fifty-one children were exposed to nevirapine for the prevention of mother-to-child HIV transmission (PMTCT) before and/or during their admissions, with additional zidovudine in 12 children.

### Serious ADRs

The multidisciplinary panel confirmed the diagnosis of 160 ADRs, 40 of which were serious (18 caused admission, 14 prolonged admission, 7 were near-fatal, and one resulted in death). Twenty serious ADRs were present at the time of admission (see Additional file 1: Table S5), and 20 occurred during admission (see Additional file 1: Table S6). The crude prevalence of serious ADRs was 3.8 per 100 drug-exposed admissions, consisting of 2.7 serious ADRs present at admission per 100 drug-exposed admissions and 1.9 serious ADRs occurring during the admission per 100 drug-exposed admissions. Alternative ways of expressing the ADR prevalence are given in the supplement (see Additional file 1: Table S7).

Thirty of 40 serious ADRs were classified as type A reactions; causality assessment rated seven type A ADRs as certain, six as probable, and 17 as possible. Ten of 40 serious ADRs were classified as type B reactions or were classified as a mixture of type A and B mechanisms. There were 17 drug-ADR pairs implicated in these ten ADRs, and causality assessment rated 2 pairs as certain, 5 as probable, and 10 as possible.

Serious ADR manifestations encountered more than once were: four cases of diarrhoea prolonging the admission, two cases of near-fatal respiratory depression, two cases of near-fatal hyperkalaemia, two cases of dystonia causing admission, two cases of urticaria causing admission, and two cases of bicytopaenia prolonging the admission.

Individual drugs most commonly implicated in serious ADRs were: prednisone (H02AB07, 5 times), methylprednisolone (H02AB04, 3 times), amoxicillin (J01CA04, 3 times), mycophenolic acid (L04AA06, 3 times), and tacrolimus (L04AD02, 3 times). Drug classes most commonly implicated in serious ADRs were (by second-level ATC code): systemic antibacterials (J01) in 12 serious ADRs, systemic corticosteroids (H02) in 6 serious ADRs, psycholeptics (N05) in 4 serious ADRs, immunosuppressants (L04) in 4 serious ADRs, direct-acting antivirals (J05) in 4 serious ADRs, and analgesics (N02) in 3 serious ADRs (Table 4). Relative to the frequency of exposure to these drug classes, immunosuppressants (L04) were disproportionately frequently implicated (see Additional file 1: Figure S1).

**Table 4:** Drug classes commonly implicated in serious ADRs and the associated ADR manifestations

Drug class	Serious ADRs
Systemic antibacterials (J01), implicated in 12 serious ADRs	• Diarrhoea with amoxicillin (2 cases) and with clarithromycin (1 case), prolonging the admissions • Urticaria with phenoxymethylpenicillin (1 case) and with ceftriaxone (1 case), causing the admissions • Maculo-papular rash with flucloxacillin, prolonging the admission • Respiratory arrest with intravenous benzathine benzylpenicillin (medication error), fatal • Red-man syndrome with vancomycin, near-fatal • Thrombocytopaenia with amoxicillin, causing the admission • Agranulocytosis with co-trimoxazole (and zidovudine), causing the admission • Bicytopaenia with ceftriaxone, co-trimoxazole (and ganciclovir), prolonging the admission • Metabolic acidosis with amikacin (and paracetamol), causing the admission
Systemic corticosteroids (H02), implicated in 6 serious ADRs	• Lower respiratory tract infection with prednisone, causing the admission • Upper respiratory tract infection with prednisone, causing the admission • *Pneumocystis jirovecii* pneumonia with prednisone and methylprednisolone, prolonging the admission • Salmonellosis with prednisone (and mycophenolic acid), prolonging the admission • Pancreatitis with prednisone and methylprednisolone, prolonging the admission • Sepsis with methylprednisolone (and mycophenolic acid and tacrolimus), prolonging the admission
Psycholeptics (N05), implicated in 4 serious ADRs	• Respiratory depression with chlorpromazine, lorazepam and diazepam (and phenobarbital), near-fatal • Respiratory depression with diazepam (and morphine and phenobarbital), near-fatal • Apnoea with midazolam, near-fatal • Delirium with clozapine, causing the admission
Immunosuppressants (L04), implicated in 4 serious ADRs	• Macrocytic anaemia with mycophenolic acid and tacrolimus, causing the admission • Neutropaenic sepsis with tacrolimus, prolonging the admission • Sepsis with mycophenolic acid and tacrolimus (and methylprednisolone), prolonging the admission • Salmonellosis with mycophenolic acid (and prednisone), prolonging the admission
Direct-acting antivirals (J05), implicated in 4 serious ADRs	• Agranulocytosis with zidovudine (and co-trimoxazole), causing the admission • Diarrhoea with lopinavir-ritonavir, prolonging the admission • Increased transaminases with efavirenz, prolonging the admission • Bicytopaenia with ganciclovir (and ceftriaxone and co-trimoxazole), prolonging the admission
Analgesics (N02), implicated in 3 serious ADRs	• Respiratory depression with morphine (and diazepam and phenobarbital), near-fatal • Metabolic acidosis with paracetamol (and amikacin), causing the admission • Increased paracetamol plasma concentration, prolonging the admission

*ADR* adverse drug reaction

Five of 30 (17%) type A serious ADRs and 4/17 (24%) type B serious ADR drug-event pairs were preventable; in total, 9/40 (23%) serious ADRs had at least one preventability factor present. The most common preventability factor was an inappropriate drug choice, which occurred in 6/40 (15%) serious ADRs. A wide variety of drugs were considered inappropriate in these cases, including benzathine benzylpenicillin, flucloxacillin, ceftriaxone, ferrous gluconate, clozapine, and amitriptyline. Inappropriate dose or route of administration occurred in 2/40 (5%) serious ADRs (drugs involved were vancomycin and metoclopramide), problems with patient adherence occurred in 2/40 (5%) serious ADRs (drugs involved were flucloxacillin and amitriptyline), insufficient laboratory monitoring occurred in 1/40 (2.5%) serious ADR attributed to tacrolimus, and a raised drug concentration occurred in 1/40 (2.5%) serious ADR attributed to tacrolimus. Alternative ways of reporting the proportion considered preventable are presented in the supplement (see Additional file 1: Table S8).

Among the 36 children with serious ADRs, one death occurred, and this was considered to be directly caused by an ADR which was the result of an error: benzathine benzylpenicillin (instead of benzylpenicillin sodium) was administered intravenously to a preterm neonate, resulting in fatal respiratory arrest. Six children with serious ADRs remained in hospital at the end of our observation period, while 29 were discharged or transferred out. The median (IQR) length of stay observed among children with serious ADRs was 6 [3–12] days. Six serious ADRs present at the time of admission were managed entirely in the short-stay ward: two children with urticaria, two children with dystonia, and one child each with lower respiratory tract infection and convulsion. Five serious ADRs occurring during the hospitalisation prolonged the hospital stay, yet the affected children were still only managed in the short-stay ward, including three children with antibiotic-associated diarrhoea, and one child each with rash and raised transaminases.

No serious ADRs occurred among term neonates. For the multivariate logistic regression analysis, we therefore grouped term neonates together with infants. The logistic regression model (Table 5) confirmed the following independent associations with serious ADRs: preterm neonates, adjusted odds ratio (aOR) with 95% CI 5.97 (1.30 to 27.3) versus referent category of infants and term neonates; middle childhood, aOR 3.63 (1.24 to 10.6) versus infants and term neonates; HIV-infection, aOR 3.87 (1.14 to 13.2) versus HIV-negative; and increasing drug count, aOR 1.08 (1.04 to 1.12) per additional drug.

**Table 5:** Multivariate logistic regression model of factors associated with serious ADR (*n* = 1001 first admissions with documented exposure to ≥1 drug(s))

	*n*	Bivariate analysis		Multivariate analysis	
		Unadjusted OR (95% CI)	*P*	Adjusted^a^ OR (95% CI)	*P*
Age category^b^					
Preterm neonate	31	5.14 (1.34 to 19.7)	0.017	5.97 (1.30 to 27.3)	0.021
Term neonate & Infant	490	Referent			
Toddler	137	1.07 (0.292 to 3.96)	0.914	1.13 (0.295 to 4.33)	0.858
Early childhood	188	1.58 (0.567 to 4.42)	0.381	1.61 (0.521 to 4.97)	0.408
Middle childhood	114	3.62 (1.40 to 9.40)	0.008	3.63 (1.24 to 10.6)	0.018
Early adolescent	41	3.79 (1.00 to 14.4)	0.050	2.88 (0.667 to 12.5)	0.156
Sex					
Male	564	Referent			
Female	437	1.08 (0.537 to 2.16)	0.832	0.894 (0.428 to 1.87)	0.766
Hospital					
RXWMCH	812	Referent			
RMMCH	189	0.953 (0.388 to 2.34)	0.917	0.938 (0.324 to 2.71)	0.906
HIV category					
Negative	840	Referent			
Infected	29	5.44 (1.76 to 16.9)	0.003	3.87 (1.14 to 13.2)	0.031
Indeterminate	132	1.34 (0.502 to 3.57)	0.560	1.59 (0.502 to 5.05)	0.429
Total drug count					
Per additional drug	1001	1.09 (1.05 to 1.13)	< 0.001	1.08 (1.04 to 1.12)	< 0.001

^a^Adjusted for other variables in the model

^b^Preterm neonate, born before 37 completed weeks’ gestation; term neonate, from birth to 27 days; infancy, 28 days to 12 months; toddler, 13 months to 24 months; early childhood, 25 months to 5 years; middle childhood, 6 years to 11 years; early adolescence, 12 years to 18 years

*ADR* adverse drug reaction; *OR* odds ratio; *RCWMCH* Red Cross War Memorial Children’s Hospital; *RMMCH* Rahima Moosa Mother and Child Hospital

In an exploratory logistic regression model (see Additional file 1: Table S9) weight-for-age z-score was not associated with serious ADR occurrence after adjustment for age, sex, hospital, HIV category, and drug count.

Four of 29 (13.8%) HIV-infected children in our survey experienced a serious ADR, including two who were admitted with serious ADRs present at the time of admission and another two who experienced serious ADRs during their admission. In three of the four cases, antiretroviral agents were implicated in the ADRs. None of the serious ADRs in HIV-infected children was considered preventable. The use of PMTCT was not implicated in any serious ADRs.

### Non-serious ADRs

We found a further 120 non-serious ADRs, which we describe in the supplement. These included 26 non-serious ADRs present at the time of admission (see Additional file 1: Table S10) and 94 non-serious ADRs occurring during the admission (see Additional file 1: Table S11). The most commonly implicated drug classes implicated in non-serious ADRs were systemic antibacterials (J01) in 48 ADRs, drugs for obstructive airway diseases (R03) in 23 ADRs, diuretics (C03) in 17 ADRs, mineral supplements (A12) in 11 ADRs, and systemic corticosteroids (H02) in 10 ADRs (see Additional file 1: Table S12).

## Discussion

In two South African children’s hospitals we found that 3.8 serious ADRs occurred per 100 drug-exposed admissions. Serious ADRs were associated with increasing drug exposure, HIV infection, and two age categories – premature neonates and middle childhood. A wide range of ADR manifestations occurred, and commonly implicated drug classes included antimicrobials, systemic corticosteroids, and antiviral agents. Considering that one in five serious ADRs was fatal or near-fatal, and about one in five serious ADRs was also preventable, these findings have significant public health importance.

It is difficult to compare the prevalence of ADRs across studies, due to differences in study settings and study designs. Nevertheless, a comprehensive 2012 systematic review [4] estimated the proportion of paediatric admissions caused by ADRs to be 2.9% (95% CI 2.6 to 3.1%), using a denominator of all admissions and not only drug-exposed admissions. In our survey 1.8% of admissions were due to an ADR. However, our survey excluded admissions to the oncology ward, whereas the 2012 systematic review’s figure is significantly influenced by two large surveys which found ADR-related admissions to oncology wards to be common [17, 18]. Published subsequent to the 2012 systematic review [4], surveys from paediatric settings in sub-Saharan Africa suggested the proportion of admissions due to ADRs to be 5.7% (16/282) in Cape Town, South Africa [19], 4.7% (114/2433) in Eritrea [20], and 0.60% (12/2004) in Lagos, Nigeria [21]. A study from Jimma, Ethiopia, with paediatric adverse drug events as outcome (i.e., a slightly different outcome than ADRs), determined the proportion of admissions to be adverse drug event-related as 0.63% (4/634) [22]. The prevalence of serious ADRs during paediatric admissions was not studied in the 2012 systematic review, although the prevalence of all in-hospital ADRs (i.e., of any seriousness) ranged from 0.6 to 16.8% among included studies [4]. An earlier systematic review found that 7 to 20% of in-hospital paediatric ADRs were serious [23], with our study’s proportion (20/114, 18%) falling within this range. A previous study from Nigeria found that 0.29% (11/3821) of retrospectively reviewed paediatric admissions were prolonged due to serious ADRs [24]. Our methodology, involving prospective review, probably explains why we found a much higher proportion of admissions (1.8%) being affected by serious ADRs occurring during the admission. Taking all these factors in consideration, our interpretation is that serious ADRs probably occurred at a similar frequency in our hospitals as in other paediatric settings in sub-Saharan Africa and elsewhere.

There is robust evidence from previous adult and paediatric systematic reviews [4, 23, 25] confirming the association between ADR occurrence and increasing drug count. Although an association between serious ADR occurrence and sex was found in the majority of studies included in the 2012 paediatric systematic review [4], we did not find any such association in this survey. Regarding associations with age, the association between ADR occurrence and prematurity which is attributable to reduced drug metabolism and clearance is also well established [26]. Previous hospital-based surveys in the United Kingdom [18, 27], United States [17], and in sub-Saharan Africa [20] have also, similar to our finding, described an association between ADR occurrence and later childhood. This association has been explained by older children’s ability to communicate their ADRs better than younger children [27]. Another explanation could be that older children tend to be admitted for chronic disease processes requiring chronic medications with a greater exposure-time within which ADRs might occur, whereas younger children are mostly admitted for acute infectious disease processes requiring short-term treatment. HIV infection causes chronic immune stimulation, increased oxidative stress, and altered patterns of drug metabolism [28], which may explain our finding that HIV infection was independently associated with the occurrence of serious ADRs. Our group has previously shown the same independent association between HIV infection and serious ADRs in South African adults [10, 29], and an association between HIV infection and serious ADRs was also found in a recent paediatric survey from another Cape Town hospital [19]. Lastly, despite our unadjusted analysis suggesting an association between serious ADR occurrence and low weight-for-age, no such association was found after adjustment for other factors.

In the 2012 systematic review, between 7 and 98% of ADRs were reportedly preventable [4]. Our proportion of serious ADRs considered preventable, i.e. 23%, is considerably lower than the findings in our adult serious ADR surveys, where 43 to 45% of ADRs were considered preventable [10, 29], but is in keeping with proportions from Nigeria (20%) [21] and Ethiopia (33%) [22]. The most common reason why serious ADRs were considered preventable in this survey was an inappropriate choice of drug (15%), which was also the most common reason in our adult surveys [10, 29] and one of the most common reasons (23%) cited in the 2012 systematic review [4]. Our survey did not yield a clear pattern of specific drugs considered inappropriate, with six different drugs involved in the six serious ADRs considered preventable for this reason.

In our survey, one child died due to an ADR, and this ADR was related to a medication error. Medication errors are common in sub-Saharan African hospitals: 75% of children admitted to general paediatric wards in a South African [30] and an Ethiopian [31] hospital were exposed to medication errors, as were 95% of children admitted to a South African paediatric intensive care unit [32]. High mortality and an association with medication error was also seen in the Ethiopian adverse drug event survey [22], where 9% of adverse drug events resulted in permanent harm or death, with three of the four events that resulted in permanent harm in that survey being due to medication error. Higher mortality rates than ours occurred in Eritrea, where 19/114 (17%) children admitted for ADRs died due to their ADRs [20], and in Nigeria, where 2/12 (17%) of children admitted for ADRs died due to their ADRs [21]. While our fatality rate may seem reassuringly low against these sub-Saharan African studies, it should be noted that an additional seven ADRs in our survey were considered near-fatal, meaning that 20% of serious ADRs may potentially have resulted in children’s deaths.

The drug classes most commonly implicated in serious ADRs in our survey were systemic antibacterials, systemic corticosteroids, antivirals, psycholeptics, immunosuppressants, and analgesics. These mostly correspond with the findings of the 2012 systematic review [4], bearing in mind we did not survey oncology wards and thus did not observe ADRs attributable to cytotoxics. Antibacterial agents were also commonly implicated in other sub-Saharan African surveys [19, 21, 22], particularly in association with rashes [21]. Significantly, our list of commonly implicated drugs includes antivirals, which was not a common cause of ADRs in the 2012 systematic review [4], and which, among three sub-Saharan African studies [19, 21, 22] was only mentioned as a commonly implicated class of drugs in one [19].

Despite choosing sites with large paediatric ART clinics, where we expected to find admissions of HIV-infected children to be concentrated, our survey included a relatively low number of HIV-infected children among those admitted. We interpret this as evidence of the efficacy of PMTCT, which has resulted in decreasing paediatric HIV incidence and prevalence [2], and of the generally high efficacy and low toxicity associated with current paediatric antiretroviral regimens in use. Nevertheless, among the small number of HIV-infected children admitted during our survey, a high proportion (9/29, 31%) had ADRs, which is comparable to another South African survey in which 9/21 (43%) HIV-infected children admitted to hospital had ADRs [19]. Our survey included 4/29 (14%) HIV-infected children with serious ADRs, of which three were attributed to the use of ART, illustrating the importance of continued vigilance when using these drugs.

One limitation of our study is that we have likely under-estimated ADR prevalence at the time of admission, as one in five folders were missing pre-admission drug exposure history, and we did not interview patients or caregivers to complement information not documented in clinical notes or verify the accuracy of recorded drug histories. Our choice of study wards (i.e., excluding surgical and oncology wards) also resulted in under-ascertainment of ADRs associated with the use of anaesthetic and cytotoxic agents, which were major drivers of serious ADRs elsewhere [17, 18, 27]. On the other hand, our study was strengthened by the inclusion of patients admitted to the short-stay ward, a setting that is often excluded [17–19, 22] from ADR surveys. We found that one quarter of all serious ADRs occurred in the short-stay ward and would have been missed had we not surveyed patients admitted there. Our study’s representativeness was also strengthened by reviewing some, although admittedly not all, neonatal admissions, and by reviewing admissions to the intensive care unit.

A strength of our study was the development of a localised trigger tool to assist in identifying potential ADRs.

We did not determine the contribution of off-label use of drugs to the burden of ADRs. This was previously described as a risk factor for development of ADRs: in the 2012 systematic review [4] three out of three studies which investigated it confirmed unlicensed or off-label use to be a risk factor for the occurrence of ADRs.

Our study was conducted in specific wards of two urban hospitals in the two best-resourced provinces of South Africa, and findings may therefore be considered context-specific, rather than generalisable to other settings. For this reason, replicating this survey in other wards, or in more resource-constrained or rurally located hospitals, and/or periodically repeating this survey or a scaled version thereof would provide a clearer picture of the burden of serious adverse drug reactions our children face.

## Conclusions

Serious ADR prevalence at two paediatric hospitals in South Africa was, at 3.8 serious ADRs per 100 drug-exposed admissions, similar to the prevalence described in hospital settings elsewhere. Similar to other sub-Saharan African studies, a large proportion of serious ADRs were fatal or near-fatal, and around one-fifth of serious ADRs were preventable. In keeping with South Africa’s high burden of HIV, we found that the antiviral drug class was one of the most commonly implicated classes in serious ADRs. Serious ADRs were independently associated with increasing drug count, HIV-infection, and two age categories: premature neonates and middle childhood.

## Supplementary information

**Additional file 1: Table S1.** Trigger tool development: summary of included studies. **Table S2.** Reasons for admission at two children’s hospitals, South Africa, 2015. **Table S3.** Background drugs most commonly in use among 1106 admissions at two children’s hospitals in South Africa, 2015. **Table S4.** Drugs most commonly in use during 1105 admissions at two children’s hospitals in South Africa, 2015. **Table S5.** Serious adverse drug reactions present at the time of admission at two children’s hospitals, South Africa, 2015. **Table S6.** Serious adverse drug reactions occurring during hospital admission at two children’s hospitals, South Africa, 2015. **Table S7.** Alternative ways of expressing ADR prevalence rates at two children’s hospitals, South Africa, 2015. **Table S8.** Reasons adverse drug reactions were considered preventable at two children’s hospitals, South Africa, 2015. **Table S9.** Exploratory analysis: Multivariate logistic regression model of factors associated with serious ADR incorporating weight-for-age z-scores, at two children’s hospitals in South Africa, 2015. **Table S10.** Non-serious adverse drug reactions present at the time of admission at two children’s hospitals, South Africa, 2015. **Table S11.** Non-serious adverse drug reactions occurring during admission at two children’s hospitals, South Africa, 2015. **Table S12.** Drug classes implicated in non-serious adverse drug reactions at two children’s hospitals, South Africa, 2015. **Figure S1.** Scatterplot of drug classes implicated in serious ADRs versus frequency of their use at two children’s hospitals, South Africa, 2015

## Data Availability

The anonymised datasets used in this study are available from the corresponding author on reasonable request.

## Abbreviations

ADR(s): Adverse drug reaction(s)
aOR: Adjusted odds ratio
ART: Antiretroviral treatment
ATC: Anatomical therapeutic chemical classification system
CI: Confidence interval
HIV: Human immunodeficiency virus
IQR: Interquartile range
NRTI: Nucleoside reverse transcriptase inhibitor
PMTCT: Prevention of mother-to-child transmission
Q1: First quartile
Q3: Third quartile
RCWMCH: Red Cross War Memorial Children’s Hospital
RMMCH: Rahima Moosa Mother and Child Hospital
WHO: World Health Organization
