# Supplementary material for: Serious adverse drug reactions at two children’s hospitals in South Africa
Source: BMC Pediatr. 2020 Jan 4;20:3. doi: 10.1186/s12887-019-1892-x (PMC6942333; doi:10.1186/s12887-019-1892-x)
Supplement: Supplementary file 1 — Additional file 1: Table S1. Trigger tool development: summary of included studies. Table S2. Reasons for admission at two children’s hospitals, South Africa, 2015. Table S3. Background drugs most commonly in use among 1106 admissions at two children’s hospitals in South Africa, 2015. Table S4. Drugs most commonly in use during 1105 admissions at two children’s hospitals in South Africa, 2015. Table S5. Serious adverse drug reactions present at the time of admission at two children’s hospitals, South Africa, 2015. Table S6. Serious adverse drug reactions occurring during hospital admission at two children’s hospitals, South Africa, 2015. Table S7. Alternative ways of expressing ADR prevalence rates at two children’s hospitals, South Africa, 2015. Table S8. Reasons adverse drug reactions were considered preventable at two children’s hospitals, South Africa, 2015. Table S9. Exploratory analysis: Multivariate logistic regression model of factors associated with serious ADR incorporating weight-for-age z-scores, at two children’s hospitals in South Africa, 2015. Table S10. Non-serious adverse drug reactions present at the time of admission at two children’s hospitals, South Africa, 2015. Table S11. Non-serious adverse drug reactions occurring during admission at two children’s hospitals, South Africa, 2015. Table S12. Drug classes implicated in non-serious adverse drug reactions at two children’s hospitals, South Africa, 2015. Figure S1. Scatterplot of drug classes implicated in serious ADRs versus frequency of their use at two children’s hospitals, South Africa, 2015 [file 12887_2019_1892_MOESM1_ESM.pdf]

Additional File 1

Serious adverse drug reactions at two children's hospitals in South Africa

Johannes P Mouton[1], Melony C Fortuin-de Smidt[1], Nicole Jobanputra[1], Ushma Mehta[2], Annemie Stewart[1], Reneé de Waal[2], Karl-Günter Technau[3], Andrew Argent[4], Max Kroon[5], Christiaan Scott[4], Karen Cohen*[1]

1. Division of Clinical Pharmacology, Department of Medicine, University of Cape Town, South Africa
2. Centre for Infectious Disease Epidemiology and Research, School of Public Health and Family Medicine, University of Cape Town, South Africa
3. Empilweni Services and Research Unit, Rahima Moosa Mother and Child Hospital, Department of Paediatrics and Child Health, School of Clinical Medicine, Faculty of Health Sciences, University of Witwatersrand, South Africa
4. Department of Paediatrics and Child Health, University of Cape Town, South Africa
5. Division of Neonatology, Department of Paediatrics and Child Health, University of Cape Town, South Africa

*Corresponding author: Associate Professor Karen Cohen, karen.cohen@uct.ac.za

Contents

Supplementary Table S1. Trigger tool development: summary of included studies.......................... 3

Supplementary Table S2. Reasons for admission at two children's hospitals, South Africa, 2015. ... 5

Supplementary Table S3. Background drugs most commonly in use among 1106 admissions at two children's hospitals in South Africa, 2015. 8

Supplementary Table S4. Drugs most commonly in use during 1105 admissions at two children's hospitals in South Africa, 2015. 9

Supplementary Table S5. Serious adverse drug reactions present at the time of admission at two children's hospitals, South Africa, 2015. 10

Supplementary Table S6. Serious adverse drug reactions occurring during hospital admission at two children's hospitals, South Africa, 2015. 11

Supplementary Table S7. Alternative ways of expressing ADR prevalence rates at two children's hospitals, South Africa, 2015 12

Supplementary Figure S1. Scatterplot of drug classes implicated in serious ADRs versus frequency of their use at two children's hospitals, South Africa, 2015 13

Supplementary Table S8. Reasons adverse drug reactions were considered preventable at two children's hospitals, South Africa, 2015. 14

Supplementary Table S9. Exploratory analysis: Multivariate logistic regression model of factors associated with serious ADR incorporating weight-for-age z-scores, at two children's hospitals in South Africa, 2015. 15

Supplementary Table S10. Non-serious adverse drug reactions present at the time of admission at two children's hospitals, South Africa, 2015. 16

Supplementary Table S11. Non-serious adverse drug reactions occurring during admission at two children's hospitals, South Africa, 2015. 17

Supplementary Table S12. Drug classes implicated in non-serious adverse drug reactions at two children's hospitals, South Africa, 2015. 21

Supplementary Table S1. Trigger tool development: summary of included studies.

Ref	Author, year	Country	Main outcome	Adults / Children	Study setting	Development of tool	Intended for use by	No. of triggers
1	Jha, 1998	USA	ADE	Adults	Surgical, medical and ICU wards	ADE detection rules, based on combinations of simple medical conditions. Modified rules with low positive predictive value.	Computer	52
2	Rozich, 2003	USA	ADE	Adults & children	86 hospitals	Expert panel, pilot testing and refining of tool.	Human	24
3	Mehta, 2007	SA	ADR	Adults	Medical wards	Additional triggers were added to increase sensitivity of the TT but method not defined.	Human	50
4	Hwang, 2008	South Korea	ADE	Adults	Surgical, medical and ICU wards	Expert panel selected and modified triggers to local clinical practices.	Computer	46
5	Franklin, 2010	UK	ADE	Adults & ?children	Surgical wards	Expert panel commented and approved triggers.	Human	23
6	Carnevali, 2013	Belgium	ADE	Adults	All admissions	Adapted to Belgian setting but method not mentioned.	Human	19
7	Sharek, 2006	USA, Canada	Harm / AE	Children*	15 NICUs	Expert panel developed list of AEs relevant to NICU, Delphi method, pilot, refined based on positive predictive value.	Human	17
8	Larsen, 2007	USA	AE	Children	1 PICU	NR	Human	25
9	Takata, 2008	USA	ADE	Children	12 paediatric hospitals	Used Rozich[2] in children, modified triggers based on positive predictive value, triggers added *a priori*.	Human	15
10	Sikdar, 2010	Canada	ADE	Children	1 emergency department	NR	Human	37
11	Agarwal, 2010	USA	AE and ADE	Children	15 PICUs	Expert panel (10) compiled TT from references and personal experience. Modified TT based on Delphi method and low positive predictive value.	Human	22
12	Matlow, 2011	Canada	AE	Children	3 acute care paediatric units (surgical and medical)	Literature review found 5 TT, compiled list. Expert panel used Delphi method to modify list.	Human	35
13	Lemon, 2012	USA	AE	Children	1 paediatric hospital	Trigger must be electronically identifiable, frequency of trigger manageable, favourable positive predictive value, significant level of harm, not investigated by other teams.	Computer	14
14	Chapman, 2014	UK	AE	Children	25 hospitals, 9 paediatric hospitals	Expert panel discussion compiled TT based on reference TT, local AEs, personal experience.	Computer	40
15	Call, 2014	USA	ADE	Children	1 hospital - oncology, hematology and catastrophic diseases	Selected triggers based on the wide use in paediatric population.	Computer	6
16	Mouton, 2016	SA	ADR	Adults	4 hospitals, medical wards	NR	Human	59

* neonates

ADE: adverse drug event; ADR: adverse drug reaction; AE: adverse event; ICU: intensive care unit; NICU: neonatal intensive care unit; NR: not reported; PICU: paediatric intensive care unit; SA: South Africa; TT: trigger tool; UK: United Kingdom; USA: United States of America

References:

1. Jha AK, Kuperman GJ, Teich JM, Leape L, Shea B, Rittenberg E, et al. Identifying Adverse Drug Events. *J Am Med Informatics Assoc.* 1998;5(3):305–14.
2. Rozich JD, Haraden CR, Resar RK. Adverse drug event trigger tool: a practical methodology for measuring medication related harm. *Qual Saf Health Care.* 2003;12(3):194–200.
3. Mehta U, Durrhelm DN, Blockman M, Kredo T, Gounden R, Barnes KI. Adverse drug reactions in adult medical inpatients in a South African hospital serving a community with a high HIV/AIDS prevalence: a prospective observational study. *Br J Clin Pharmacol.* 2007; 65(3):396-406.
4. Hwang S-H, Lee S, Koo H-K, Kim Y. Evaluation of a computer-based adverse-drug-event monitor. *Am J Health Syst Pharm.* 2008;65(23):2265–72.
5. Franklin BD, Birch S, Schachter M, Barber N. Testing a trigger tool as a method of detecting harm from medication errors in a UK hospital: a pilot study. *Int J Pharm Pract.* 2010;18(5):305–11.
6. Carnevali L, Krug B, Amant F, Van Pee D, Gérard V, de Béthune X, et al. Performance of the adverse drug event trigger tool and the global trigger tool for identifying adverse drug events: experience in a Belgian hospital. *Ann Pharmacother.* 2013;47(11):1414–9.
7. Sharek PJ, Horbar JD, Mason W, Bisarya H, Thurm CW, Suresh G, et al. Adverse events in the neonatal intensive care unit: development, testing, and findings of an NICU-focused trigger tool to identify harm in North American NICUs. *Pediatrics.* 2006;118(4):1332–40.
8. Larsen GY, Donaldson AE, Parker HB, Grant MJC. Preventable harm occurring to critically ill children. *Pediatr Crit Care Med.* 2007;8(4):331–6.
9. Takata GS, Mason W, Taketomo C, Logsdon T, Sharek PJ. Development, testing, and findings of a pediatric-focused trigger tool to identify medication-related harm in US children's hospitals. *Pediatrics.* 2008;121(4):e927–e935.
10. Sikdar KC, Alaghehbandan R, Macdonald D, Barrett B, Collins KD, Gadag V. Adverse drug events among children presenting to a hospital emergency department in Newfoundland and Labrador, Canada. Pharmacoepidemiol *Drug Saf.* 2010;19(2):132–40.
11. Agarwal S, Classen D, Larsen G, Tofil NM, Hayes LW, Sullivan JE, et al. Prevalence of adverse events in pediatric intensive care units in the United States. *Pediatr Crit Care Med.* 2010;11(5):568–78.
12. Matlow AG, Cronin CMG, Flintoft V, Nijssen-Jordan C, Fleming M, Brady-Fryer B, et al. Description of the development and validation of the Canadian Paediatric Trigger Tool. *BMJ Qual Saf.* 2011;20(5):416–23.
13. Lemon V, Stockwell DC. Automated Detection of Adverse Events in children. *Pediatr Clin N Am.* 2012, 59:1269-1278.
14. Chapman SM, Fitzsimons J, Davey N, Lachman P. Prevalence and severity of patient harm in a sample of UK-hospitalised children detected by the Paediatric Trigger Tool. *BMJ Open.* 2014;4(7):e005066.
15. Call RJ, Burlison JD, Robertson JJ, Scott JR, Baker DK, Rossi MG, et al. Adverse drug event detection in pediatric oncology and hematology patients: using medication triggers to identify patient harm in a specialized pediatric patient population. *J Pediatr.* 2014;165(3):447–52.e4.
16. Mouton JP, Njuguna C, Kramer N, et al. Adverse Drug Reactions Causing Admission to Medical Wards: A Cross-Sectional Survey at 4 Hospitals in South Africa. *Medicine (Baltimore)*. 2016;95(19):e3437.

Supplementary Table S2. Reasons for admission at two children's hospitals, South Africa, 2015.

ICD-10 Chapter / Code block	Count	Proportion
**CERTAIN INFECTIOUS AND PARASITIC DISEASES**	**140**	**8.7%**
A00-A09 Intestinal infectious diseases	56	3.5%
A15-A19 Tuberculosis	19	1.2%
A30-A49 Other bacterial diseases	30	1.9%
A50-A64 Infections with a predominantly sexual mode of transmission	1	0.06%
B00-B09 Viral infections characterized by skin and mucous membrane lesions	9	0.56%
B15-B19 Viral hepatitis	3	0.19%
B20-B24 Human immunodeficiency virus [HIV] disease	4	0.25%
B25-B34 Other viral diseases	4	0.25%
B35-B49 Mycoses	10	0.62%
B50-B64 Protozoal diseases	1	0.06%
B65-B83 Helminthiases	1	0.06%
B85-B89 Pediculosis, ascariasis and other infestations	2	0.12%
**NEOPLASMS**	**10**	**0.62%**
C64-C68 Malignant neoplasms of urinary tract	1	0.06%
C69-C72 Malignant neoplasms of eye, brain and other parts of the central nervous system	2	0.12%
D10-D36 Benign neoplasms	5	0.31%
D37-D48 Neoplasms of uncertain or unknown behaviour	2	0.12%
**DISEASES OF THE BLOOD AND BLOOD-FORMING ORGANS; CERTAIN DISORDERS INVOLVING THE IMMUNE MECHANISM**	**81**	**5.1%**
D50-D53 Nutritional anaemias	25	1.6%
D55-D59 Haemolytic anaemias	3	0.19%
D60-D64 Aplastic and other anaemias	33	2.1%
D65-D69 Coagulation defects, purpura and other haemorrhagic conditions	5	0.31%
D70-D77 Other diseases of blood and blood-forming organs	14	0.87%
D80-D89 Certain disorders involving the immune mechanism	1	0.06%
**ENDOCRINE, NUTRITIONAL AND METABOLIC DISEASES**	**97**	**6.1%**
E00-E07 Disorders of thyroid gland	2	0.12%
E10-E14 Diabetes mellitus	2	0.12%
E15-E16 Other disorders of glucose regulation and pancreatic internal secretion	3	0.19%
E20-E35 Disorders of other endocrine glands	4	0.25%
E40-E46 Malnutrition	31	1.9%
E50-E64 Other nutritional deficiencies	2	0.12%
E70-E90 Metabolic disorders	53	3.3%
**MENTAL AND BEHAVIOURAL DISORDERS**	**4**	**0.25%**
F00-F09 Organic, including symptomatic, mental disorders	1	0.06%
F70-F79 Mental retardation	1	0.06%
F80-F89 Disorders of psychological development	2	0.12%
**DISEASES OF THE NERVOUS SYSTEM**	**60**	**3.7%**
G00-G09 Inflammatory diseases of the central nervous system	22	1.4%
G10-G14 Systemic atrophies primarily affecting the central nervous system	2	0.12%
G20-G26 Extrapyramidal and movement disorders	5	0.31%
G35-G37 Demyelinating diseases of the central nervous system	1	0.06%
G40-G47 Episodic and paroxysmal disorders	21	1.3%
G50-G59 Nerve, nerve root and plexus disorders	1	0.06%
G60-G64 Polyneuropathies and other disorders of the peripheral nervous system	1	0.06%
G80-G83 Cerebral palsy and other paralytic syndromes	4	0.25%
G90-G99 Other disorders of the nervous system	3	0.19%

**DISEASES OF THE EYE AND ADNEXA**	**13**	**0.81%**
H10-H13 Disorders of conjunctiva	12	0.75%
H49-H52 Disorders of ocular muscles, binocular movement, accommodation and refraction	1	0.06%
**DISEASES OF THE EAR AND MASTOID PROCESS**	**23**	**1.4%**
H65-H75 Diseases of middle ear and mastoid	23	1.4%
**DISEASES OF THE CIRCULATORY SYSTEM**	**33**	**2.1%**
I10-I15 Hypertensive diseases	4	0.25%
I26-I28 Pulmonary heart disease and diseases of the pulmonary circulation	6	0.37%
I30-I52 Other forms of heart disease	20	1.2%
I70-I79 Diseases of arteries, arterioles and capillaries	1	0.06%
I80-I89 Diseases of veins, lymphatic vessels and lymph nodes	1	0.06%
I95-I99 Other and unspecified disorders of the cardiovascular system	1	0.06%
**DISEASES OF THE RESPIRATORY SYSTEM**	**598**	**37%**
J00-J06 Acute upper respiratory infections	56	3.5%
J09-J18 Influenza and pneumonia	202	13%
J20-J22 Other acute lower respiratory infections	270	17%
J30-J39 Other diseases of upper respiratory tract	5	0.31%
J40-J47 Chronic lower respiratory diseases	37	2.3%
J80-J84 Other respiratory diseases principally affecting the interstitium	1	0.06%
J90-J94 Other diseases of pleura	2	0.12%
J95-J99 Other diseases of the respiratory system	25	1.6%
**DISEASES OF THE DIGESTIVE SYSTEM**	**40**	**2.5%**
K00-K14 Diseases of oral cavity, salivary glands and jaws	6	0.37%
K20-K31 Diseases of oesophagus, stomach and duodenum	1	0.06%
K40-K46 Diseases of appendix	1	0.06%
K50-K52 Noninfective enteritis and colitis	11	0.69%
K55-K64 Other diseases of intestines	9	0.56%
K65-K67 Diseases of peritoneum	1	0.06%
K70-K77 Diseases of liver	6	0.37%
K80-K87 Disorders of gallbladder, biliary tract and pancreas	2	0.12%
K90-K93 Other diseases of the digestive system	3	0.19%
**DISEASES OF THE SKIN AND SUBCUTANEOUS TISSUE**	**31**	**1.9%**
L00-L08 Infections of the skin and subcutaneous tissue	12	0.75%
L20-L30 Dermatitis and eczema	12	0.75%
L40-L45 Papulosquamous disorders	1	0.06%
L50-L54 Urticaria and erythema	4	0.25%
L80-L99 Other disorders of the skin and subcutaneous tissue	2	0.12%
**DISEASES OF THE MUSCULOSKELETAL SYSTEM AND CONNECTIVE TISSUE**	**9**	**0.56%**
M00-M03 Infectious arthropathies	1	0.06%
M30-M36 Systemic connective tissue disorders	4	0.25%
M60-M63 Disorders of muscles	1	0.06%
M86-M90 Other osteopathies	2	0.12%
M91-M94 Chondropathies	1	0.06%
**DISEASES OF THE GENITOURINARY SYSTEM**	**54**	**3.4%**
N00-N08 Glomerular diseases	6	0.37%
N10-N16 Renal tubulo-interstitial diseases	1	0.06%
N17-N19 Renal failure	20	1.2%
N30-N39 Other diseases of urinary system	26	1.6%
N40-N51 Disorders of male genital organs	1	0.06%

**CERTAIN CONDITIONS ORIGINATING IN THE PERINATAL PERIOD**	**85**	**5.3%**
P05-P08 Disorders related to length of gestation and foetal growth	5	0.31%
P20-P29 Respiratory and cardiovascular disorders specific to the perinatal period	10	0.62%
P35-P39 Infections specific to the perinatal period	31	1.9%
P50-P61 Haemorrhagic and haematological disorders of foetus and newborn	34	2.1%
P75-P78 Digestive system disorders of foetus and newborn	2	0.12%
P80-P83 Conditions involving the integument and temperature regulation of foetus and newborn	1	0.06%
P90-P96 Other disorders originating in the perinatal period	2	0.12%
**CONGENITAL MALFORMATIONS, DEFORMATIONS AND CHROMOSOMAL ABNORMALITIES**	**52**	**3.2%**
Q00-Q07 Congenital malformations of the nervous system	1	0.06%
Q20-Q28 Congenital malformations of the circulatory system	46	2.9%
Q38-Q45 Other congenital malformations of the digestive system	2	0.12%
Q60-Q64 Congenital malformations of the urinary system	1	0.06%
Q65-Q79 Congenital malformations and deformations of the musculoskeletal system	1	0.06%
Q80-Q89 Other congenital malformations	1	0.06%
**SYMPTOMS, SIGNS AND ABNORMAL CLINICAL AND LABORATORY FINDINGS**	**218**	**14%**
R00-R09 Symptoms and signs involving the circulatory and respiratory systems	59	3.7%
R10-R19 Symptoms and signs involving the digestive system and abdomen	17	1.1%
R20-R23 Symptoms and signs involving the skin and subcutaneous tissue	9	0.56%
R30-R39 Symptoms and signs involving the urinary system	1	0.06%
R40-R46 Symptoms and signs involving cognition, perception, emotional state and behaviour	1	0.06%
R50-R69 General symptoms and signs	122*	7.6%
R70-R79 Abnormal findings on examination of blood, without diagnosis	7	0.44%
R80-R82 Abnormal findings on examination of urine, without diagnosis	2	0.12%
**INJURY, POISONING AND CERTAIN OTHER CONSEQUENCES OF EXTERNAL CAUSES**	**28**	**1.7%**
S00-S09 Injuries to the head	7	0.44%
T08-T14 Injuries to unspecified part of trunk, limb or body region	1	0.06%
T15-T19 Effects of foreign body entering through natural orifice	1	0.06%
T20-T32 Burns and corrosions	3	0.19%
T36-T50 Poisoning by drugs, medicaments and biological substances	7	0.44%
T66-T78 Other and unspecified effects of external causes	5	0.31%
T80-T88 Complications of surgical and medical care	4	0.25%
**EXTERNAL CAUSES OF MORBIDITY AND MORTALITY**	**16**	**1.0%**
W65-W74 Accidental drowning and submersion	1	0.06%
X40-X49 Accidental poisoning by and exposure to noxious substances	12	0.75%
X85-Y09 Assault	2	0.12%
Y83-Y84 Surgery and other medical procedures as the cause of abnormal reaction of the patient, or of later complication, without mention of misadventure at the time of procedure	1	0.06%
**FACTORS INFLUENCING HEALTH STATUS AND CONTACT WITH HEALTH SERVICES**	**10**	**0.62%**
Z00-Z13 Persons encountering health services for examination and investigation	2	0.12%
Z20-Z29 Persons with potential health hazards related to communicable diseases	1	0.06%
Z40-Z54 Persons encountering health services for specific procedures and health care	4	0.25%
Z70-Z76 Persons encountering health services in other circumstances	2	0.12%
Z80-Z99 Persons with potential health hazards related to family and personal history and certain conditions influencing health status	1	0.06%
**TOTAL**	**1602**	**100%**

Reasons for admission are presented according to ICD-10 classification. More than one reason possible per admission.

* Including 93 admissions for convulsions

Supplementary Table S3. Background drugs most commonly in use among 1106 admissions at two children's hospitals in South Africa, 2015.

Drug class	ATC	Prior to *n* admissions	Frequency of use (N=1106)
Adrenergics, inhalants	R03A	281	25.41%
Other analgesics and antipyretics	N02B	277	25.05%
Beta-lactam antibacterials, penicillins	J01C	211	19.08%
Other beta-lactam antibacterials	J01D	152	13.74%
Other drugs for obstructive airway disease	R03B	125	11.30%
Corticosteroids for systemic use, plain	H02A	104	9.40%
Antiepileptics	N03A	88	7.96%
Multivitamins, combinations	A11A	80	7.23%
Direct acting antivirals	J05A	73	6.60%
Sulfonamides and trimethoprim	J01E	61	5.52%
Other mineral supplements	A12C	59	5.33%
Drugs for peptic ulcer and gastroesophageal reflux	A02B	58	5.24%
High-ceiling diuretics	C03C	58	5.24%
Vitamin B12 and folic acid	B03B	53	4.79%
Antihistamines for systemic use	R06A	47	4.25%
Vitamin A and D, incl. combinations	A11C	46	4.16%
Anxiolytics	N05B	45	4.07%
Potassium-sparing agents	C03D	44	3.98%
Aminoglycoside antibacterials	J01G	35	3.16%
Iron preparations	B03A	35	3.16%
Drugs for treatment of tuberculosis	J04A	31	2.80%
Laxatives	A06A	30	2.71%
ACE inhibitors, plain	C09A	28	2.53%
Macrolides, lincosamides and streptogramines	J01F	27	2.44%
I.V. solution additives	B05X	26	2.35%
Adrenergics for systemic use	R03C	25	2.26%
Calcium	A12A	24	2.17%
Anaesthetics, general	N01A	21	1.90%
Antibacterials (unspecified)	J01	20	1.81%
Opioids	N02A	20	1.81%

Background drug use was defined as drug use over a 30-day period before the admission. Drugs grouped by third-level ATC group.

Supplementary Table S4. Drugs most commonly in use during 1105 admissions at two children's hospitals in South Africa, 2015.

**Drug class**	**ATC**	**During *n* admissions**	**Frequency of use (N=1105)**
Other analgesics and antipyretics	N02B	667	60.36%
Beta-lactam antibacterials, penicillins	J01C	658	59.55%
Adrenergics, inhalants	R03A	416	37.65%
Other mineral supplements	A12C	314	28.42%
Other beta-lactam antibacterials	J01D	297	26.88%
Multivitamins, combinations	A11A	292	26.43%
Aminoglycoside antibacterials	J01G	214	19.37%
Corticosteroids for systemic use, plain	H02A	178	16.11%
Vitamin B12 and folic acid	B03B	156	14.12%
Potassium	A12B	149	13.48%
Vitamin A and D, incl. combinations	A11C	147	13.30%
Direct acting antivirals	J05A	136	12.31%
Antiepileptics	N03A	130	11.76%
High-ceiling diuretics	C03C	129	11.67%
Opioids	N02A	121	10.95%
Anesthetics, general	N01A	118	10.68%
I.V. solution additives	B05X	116	10.50%
Other drugs for obstructive airway disease	R03B	113	10.23%
Antiadrenergic agents, centrally acting	C02A	99	8.96%
Intestinal antiinfectives	A07A	94	8.51%
Drugs for peptic ulcer and gastroesophageal reflux	A02B	89	8.05%
Antiinflammatory and antirheumatic preparations	M01A	86	7.78%
Hypnotics and sedatives	N05C	84	7.60%
Potassium-sparing agents	C03D	79	7.15%
Macrolides, lincosamides and streptogramines	J01F	77	6.97%
Viral vaccines	J07B	69	6.24%
Sulfonamides and trimethoprim	J01E	66	5.97%
Anxiolytics	N05B	61	5.52%
Drugs for treatment of tuberculosis	J04A	58	5.25%
Calcium	A12A	56	5.07%
Laxatives	A06A	54	4.89%
Other antibacterials	J01X	53	4.80%
Cardiac stimulants excl. cardiac glycosides	C01C	51	4.62%
Vitamin K and other haemostatics	B02B	49	4.43%
Antihistamines for systemic use	R06A	48	4.34%
Antinematodal agents	P02C	45	4.07%
Iron preparations	B03A	43	3.89%
Bacterial vaccines	J07A	40	3.62%
Other plain vitamin preparations	A11H	39	3.53%
Bacterial and viral vaccines, combined	J07C	34	3.08%
Muscle relaxants, peripherally acting	M03A	34	3.08%
Blood and related products	B05A	33	2.99%
Psychostimulants	N06B	33	2.99%
Ace inhibitors, plain	C09A	32	2.90%
Antimycotics for systemic use	J02A	27	2.44%
Other systemic drugs for obstructive airways disease	R03D	25	2.26%
Quinolone antibacterials	J01M	20	1.81%

Drugs grouped by third-level ATC group.

Supplementary Table S5. Serious adverse drug reactions present at the time of admission at two children's hospitals, South Africa, 2015.

Patient	Sex	Age category	Weight (kg)	WFA-z	HIV category	ADR no.	ADR manifestation (MedDRA® PT)	Type	Implicated drug(s)	Seriousness	Causality	Preventable
1	Female	Preterm neonate	0.7		Negative	1	Neonatal respiratory depression	A	Remifentanil	Caused admission	Possible	No
2	Female	Preterm neonate	1.9		Negative	2	Cardio-respiratory arrest	A	Fentanyl	Caused admission	Possible	No
3	Male	Preterm neonate	1.5		Negative	3	Respiratory arrest	B	Benzathine benzylpenicillin	Death	Definite	Yes
4	Male	Infancy		-5.5	Indeterminate	4	Cardiogenic shock	A	Furosemide + spironolactone	Caused admission	Possible	No
5	Male	Toddler		-0.8	Negative	5	Urticaria	B	Phenoxymethylpenicillin	Caused admission	Definite	No
6	Female	Toddler		-1.3	Negative	6	Cardiac failure congestive	A	Carvedilol	Caused admission	Possible	No
7	Male	Toddler		-1.6	Positive	7	Lower respiratory tract infection	A	Prednisone	Caused admission	Possible	No
8	Male	Toddler		-2.6	Negative	8	Dystonia	A	Metoclopramide	Caused admission	Definite	No
9	Female	Early childhood		0.9	Negative	9	Urticaria	B	Ceftriaxone	Caused admission	Probable	Yes
10	Male	Early childhood		-3.1	Positive	10	Agranulocytosis	Mixed	Zidovudine	Caused admission	Probable	No
									Co-trimoxazole		Possible	No
11	Female	Early childhood	18		Negative	11	Convulsion	A	Isoniazid	Caused admission	Possible	No
12	Male	Middle childhood	31		Negative	12	Thrombocytopenia	B	Amoxicillin	Caused admission	Possible	No
13	Female	Middle childhood	Missing		Negative	13	Constipation	A	Ferrous gluconate	Caused admission	Possible	Yes
14	Female	Middle childhood	25		Negative	14	Metabolic acidosis	A	Amikacin + paracetamol	Caused admission	Possible	No
15	Female	Middle childhood	51		Negative	15	Delirium	A	Clozapine	Caused admission	Probable	Yes
16	Female	Middle childhood	74		Negative	16	Upper respiratory tract infection	A	Prednisone	Caused admission	Possible	No
17	Female	Middle childhood	28		Negative	17	Dystonia	A	Metoclopramide	Caused admission	Definite	Yes
18	Male	Early adolescence	40		Negative	18	Somnolence	A	Amitriptyline	Caused admission	Definite	Yes
19	Female	Early adolescence	42		Negative	19	Hyperkalaemia	A	Enalapril	Near-death	Definite	No
20	Female	Early adolescence	37		Negative	20	Anaemia macrocytic	Mixed	Tacrolimus	Caused admission	Possible	No
									Mycophenolic acid		Possible	No
									Erythropoietin		Possible	No

MedDRA® PT: Medical Dictionary for Regulatory Activities preferred term; WFA-z: weight-for-age z-score

Supplementary Table S6. Serious adverse drug reactions occurring during hospital admission at two children's hospitals, South Africa, 2015.

Patient	Sex	Age category	Weight (kg)	WFA-z	HIV category	ADR no.	ADR manifestation (MedDRA® PT)	Type	Implicated drug(s)	Seriousness	Causality	Preventable
21	Female	Infancy		0.5	Indeterminate	21	Respiratory depression	A	Lorazepam + chlorpromazine + diazepam + phenobarbital	Near-death	Probable	No
22	Female	Infancy		-6.8	Negative	22	Red man syndrome	B	Vancomycin	Near-death	Probable	Yes
23	Male	Infancy		-0.3	Indeterminate	23	Respiratory depression	A	Morphine + diazepam + phenobarbital	Near-death	Possible	No
24	Male	Infancy		-1.5	Indeterminate	24	Apnoea	A	Midazolam	Near-death	Possible	No
25	Male	Infancy		-1.6	Negative	25	Diarrhoea	A	Amoxicillin	Prolonged admission	Possible	No
26	Male	Infancy		-5.7	Positive	26	Diarrhoea	A	Lopinavir / ritonavir	Prolonged admission	Probable	No
27	Male	Infancy		-2.7	Negative	27	Diarrhoea	A	Clarithromycin	Prolonged admission	Possible	No
28	Male	Infancy		-0.5	Negative	28	Diarrhoea	A	Amoxicillin	Prolonged admission	Possible	No
29	Male	Infancy		-0.5	Indeterminate	29	Analgesic drug level increased	A	Paracetamol	Prolonged admission	Definite	No
						30	Bicytopenia	A	Ceftriaxone + ganciclovir + co-trimoxazole	Prolonged admission	Possible	No
30	Female	Early childhood		0.8	Negative	31	Rash maculo-papular	B	Flucloxacillin	Prolonged admission	Possible	Yes
31	Female	Early childhood		-1.3	Negative	32	Neutropenic sepsis	A	Tacrolimus	Prolonged admission	Probable	Yes
32	Female	Early childhood	23		Negative	33	Sepsis	A	Methylprednisolone + tacrolimus + mycophenolic acid	Prolonged admission	Possible	No
33	Male	Middle childhood	21		Negative	34	Hyperkalaemia	A	Enalapril	Near-death	Definite	No
						35	Renal failure acute	A	Furosemide	Near-death	Probable	No
34	Male	Middle childhood	26		Positive	36	Transaminases increased	B	Rifampicin	Prolonged admission	Possible	No
									Pyrazinamide		Possible	No
									Isoniazid		Possible	No
									Efavirenz		Possible	No
35	Female	Middle childhood	38		Negative	37	Bicytopenia	A	Cyclophosphamide	Prolonged admission	Definite	No
						38	Pancreatitis	B	Methylprednisolone	Prolonged admission	Probable	No
									Prednisone		Probable	No
						39	*Pneumocystis jirovecii* pneumonia	A	Prednisone + methylprednisolone	Prolonged admission	Possible	No
36	Male	Early adolescence	41		Negative	40	Salmonellosis	A	Mycophenolic acid + prednisone	Prolonged admission	Probable	No

MedDRA® PT: Medical Dictionary for Regulatory Activities preferred term; WFA-z: weight-for-age z-score

Supplementary Table S7. Alternative ways of expressing ADR prevalence rates at two children's hospitals, South Africa, 2015

Outcome event	(n) events	Prevalence per 100 admissions	
		Denominator: all admissions [a]	Denominator: drug-exposed admissions only [b]
ADRs	160	14.5	15.1
Serious ADRs	40	3.62	3.78
Serious ADRs present at admission	20	1.81	2.67
Serious ADR occurring during admission	20	1.81	1.93
Non-serious ADRs	120	10.8	11.4
Non-serious ADRs present at admission	26	2.35	3.47
Non-serious ADR occurring during admission	94	8.51	9.07
ADRs present at admission	46	4.16	6.13
ADRs occurring during admission	114	10.3	11.0
Admissions with ≥1 ADR	120	10.8	11.4
Admissions with ≥1 serious ADR	36	3.26	3.41
Admissions with ≥1 serious ADR present at admission	20	1.81	2.67
Admissions with ≥1 serious ADR during admission	16	1.45	1.54
Admissions with ≥1 non-serious ADR	94	8.50	8.89
Admissions with ≥1 non-serious ADR present at admission	25	2.26	3.33
Admissions with ≥1 non-serious ADR during admission	72	6.52	6.95
Admissions with ≥1 ADR present at admission	41	3.71	5.47
Admissions with ≥1 ADR during admission	85	7.69	8.20

[a] Denominator (N) = 1106 admissions for "present at admission" outcomes; N=1105 admissions for "during admission" outcomes; N=1106 admissions for "present at admission or during admission" outcomes

[b] Denominator (N) = 750 drug-exposed admissions for "present at admission" outcomes; N=1036 drug-exposed admissions for "during admission" outcomes; N=1057 drug-exposed admissions for "present at admission or during admission" outcomes

Supplementary Figure S1. Scatterplot of drug classes implicated in serious ADRs versus frequency of their use at two children's hospitals, South Africa, 2015

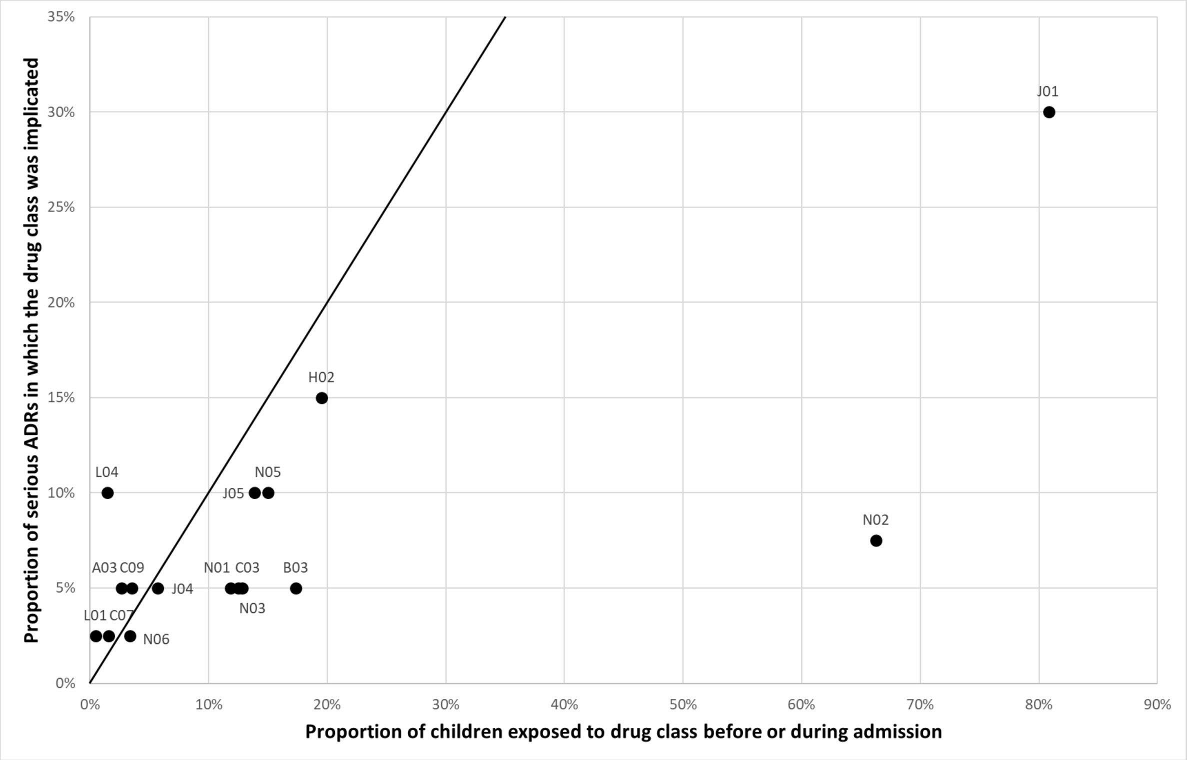

Scatterplot of 15 drug classes (coded to second-level ATC codes) implicated in 40 serious ADRs. For points above the diagonal line, drug classes were disproportionately often implicated in ADRs, compared to their frequency of exposure. A03 = drugs for functional gastrointestinal disorders; B03 = antianaemic preparations; C03 = diuretics; C07 = beta blocking agents; C09 = agents acting on renin-angiotensin system; H02 = corticosteroids for systemic use; J01 = antibacterials for systemic use; J04 = antimycobacterials; J05 = antivirals for systemic use; L01 = antineoplastic agents; L04 = immunosuppressive agents; N01 = anaesthetics; N02 = analgesics; N03 = antiepileptics; N05 = psycholeptics; N06 = psychoanaleptics

Supplementary Table S8. Reasons adverse drug reactions were considered preventable at two children's hospitals, South Africa, 2015.

	Proportion considered preventable							
	**Drug-ADR-pair-level**		**ADR-level**		**Admission-level**		**Patient-level**	
**Schumock and Thornton preventability factor**	Drug-ADR pairs with preventability factor present (N=264 pairs)	Drug-serious- ADR pairs with preventability factor present (N=60 pairs)	ADRs with preventability factor present (N=160 ADRs)	Serious ADRs with preventability factor present (N=40 ADRs)	Admissions with ≥1 ADR with preventability factor present (N=120 admissions)	Admissions with ≥1 serious ADR with preventability factor present (N=36 admissions)	Patients with ≥1 ADR with preventability factor present (N=119 patients)	Patients with ≥1 serious ADR with preventability factor present (N=36 patients)
Drug inappropriate	15 (5.7%)	6 (10%)	14 (8.8%)	6 (15%)	14 (12%)	6 (17%)	14 (12%)	6 (17%)
Dose/route inappropriate	5 (1.9%)	2 (3.3%)	5 (3.1%)	2 (5.0%)	5 (4.2%)	2 (5.6%)	5 (4.2%)	2 (5.6%)
Monitoring insufficient	8 (3.0%)	1 (1.7%)	8 (5.0%)	1 (2.5%)	8 (6.7%)	1 (2.8%)	7 (5.9%)	1 (2.8%)
History of ADR	1 (0.38%)	0	1 (0.63%)	0	1 (0.83%)	0	1 (0.84%)	0
Interaction	0	0	0	0	0	0	0	0
Raised drug concentration	2 (0.76%)	1 (1.7%)	2 (1.3%)	1 (2.5%)	2 (1.7%)	1 (2.8%)	2 (1.7%)	1 (2.8%)
Compliance	2 (0.76%)	2 (3.3%)	2 (1.3%)	2 (5.0%)	2 (1.7%)	2 (5.6%)	2 (1.7%)	2 (5.6%)
**Any factor**	**26 (9.8%)**	**9 (15%)**	**25 (16%)**	**9 (23%)**	**24 (20%)**	**9 (25%)**	**23 (19%)**	**9 (25%)**

Supplementary Table S9. Exploratory analysis: Multivariate logistic regression model of factors associated with serious ADR incorporating weight-for-age z-scores, at two children's hospitals in South Africa, 2015.

	n	Bivariate analysis		Multivariate analysis	
		**Unadjusted OR (95% CI)**	**P**	**Adjusted[a] OR (95% CI)**	**P**
**Age category**					
Infant	415	Referent			
Toddler	135	0.920 (0.250 to 3.39)	0.901	1.21 (0.295 to 4.94)	0.794
Early childhood	159	1.05 (0.323 to 3.38)	0.941	1.53 (0.391 to 5.93)	0.544
**Sex**					
Male	419	Referent			
Female	290	0.595 (0.207 to 1.71)	0.334	0.585 (0.196 to 1.74)	0.335
**Weight-for-age z-score**					
Per 1-unit increase	709	0.785 (0.623 to 0.989)	0.040	0.908 (0.687 to 1.20)	0.498
**Hospital**					
RXWMCH	589	Referent			
RMMCH	120	1.05 (0.298 to 3.72)	0.936	0.851 (0.221 to 3.28)	0.815
**HIV category**					
Negative	576	Referent			
Infected	22	9.95 (2.49 to 39.7)	0.001	6.21 (1.40 to 27.5)	0.016
Indeterminate	111	2.97 (0.977 to 9.04)	0.055	2.60 (0.737 to 9.19)	0.137
**Total drug count**					
Per additional drug	709	1.10 (1.04 to 1.15)	<0.001	1.07 (1.01 to 1.14)	0.022

n = 709 first admissions of infants, toddlers, and under-6-year-olds

[a] Adjusted for other variables in the model

Supplementary Table S10. Non-serious adverse drug reactions present at the time of admission at two children's hospitals, South Africa, 2015.

Patient	Sex	Age category	Weight (kg)	WFA-z	HIV category	ADR no.	ADR manifestation (MedDRA® PT)	Type	Implicated drug(s)	Seriousness	Causality	Preventable
37	Female	Term neonate	3.6		Indeterminate	41	Tachycardia	A	Salbutamol	Increased monitoring, no harm	Possible	No
38	Female	Infancy		-5.6	Indeterminate	42	Normochromic normocytic anaemia	A	Zidovudine	Requires treatment, temporary harm	Possible	No
39	Male	Infancy		-0.8	Indeterminate	43	Diarrhoea	A	Erythromycin	Increased monitoring, no harm	Possible	Yes
25	Male	Infancy		-1.6	Negative	44	Tachycardia	A	Epinephrine	Increased monitoring, no harm	Probable	No
40	Male	Toddler		-0.8	Negative	45	Hypokalaemia	A	Epinephrine + salbutamol	Requires treatment, temporary harm	Probable	No
41	Male	Early childhood		0.0	Negative	46	Transaminases increased	B	Co-amoxiclav	Requires treatment, temporary harm	Possible	No
42	Male	Early childhood		-1.2	Negative	47	Hypokalaemia	A	Furosemide	Increased monitoring, no harm	Possible	No
43	Male	Early childhood		-1.4	Negative	48	Bicytopenia	B	Furosemide	Increased monitoring, no harm	Possible	No
44	Female	Early childhood		0.8	Negative	49	Respiratory depression	A	Diazepam	Requires treatment, temporary harm	Possible	No
45	Male	Early childhood		0.0	Negative	50	Rash erythematous	B	Clarithromycin	Requires treatment, temporary harm	Possible	No
									Amoxicillin		Possible	Yes
46	Female	Early childhood		-0.6	Negative	51	Hallucination	A	Diphenhydramine	Increased monitoring, no harm	Possible	Yes
47	Male	Early childhood		-0.6	Negative	52	Tachycardia	A	Salbutamol + fenoterol	Increased monitoring, no harm	Probable	No
10	Male	Early childhood		-3.1	Positive	53	Hyponatraemia	A	Co-trimoxazole	Requires treatment, temporary harm	Possible	No
48	Male	Early childhood	19		Negative	54	Tachycardia	A	Salbutamol + fenoterol	Increased monitoring, no harm	Probable	No
49	Female	Early childhood	16		Negative	55	Tachycardia	A	Fenoterol	Increased monitoring, no harm	Possible	No
32	Female	Early childhood	23		Negative	56	Cushingoid	A	Methylprednisolone	Increased monitoring, no harm	Definite	No
12	Male	Middle childhood	31		Negative	57	Cushingoid	A	Prednisone	Increased monitoring, no harm	Definite	No
50	Male	Middle childhood	23		Negative	58	Tachycardia	A	Salbutamol + fenoterol	Increased monitoring, no harm	Possible	No
34	Male	Middle childhood	26		Positive	59	Hyponatraemia	A	Co-trimoxazole	Increased monitoring, no harm	Possible	No
16	Female	Middle childhood	74		Negative	60	Cushingoid	A	Prednisone	Increased monitoring, no harm	Definite	No
51	Male	Middle childhood	33		Negative	61	Hypokalaemia	A	Salbutamol + fenoterol	Requires treatment, temporary harm	Probable	No
						62	Tachycardia	A	Salbutamol + fenoterol	Increased monitoring, no harm	Probable	No
52	Male	Early adolescence	33		Negative	63	Somnolence	A	Phenobarbital	Increased monitoring, no harm	Definite	Yes
53	Female	Early adolescence	22		Negative	64	Constipation	A	Atropine	Requires treatment, temporary harm	Possible	No
19	Female	Early adolescence	42		Negative	65	Pruritus	B	Heparin	Increased monitoring, no harm	Definite	No
						66	Hypokalaemia	A	Polystyrene sulfonate	Increased monitoring, no harm	Definite	Yes

MedDRA® PT: Medical Dictionary for Regulatory Activities preferred term; WFA-z: weight-for-age z-score

Supplementary Table S11. Non-serious adverse drug reactions occurring during admission at two children's hospitals, South Africa, 2015.

Patient	Sex	Age category	Weight (kg)	WFA-z	HIV category	ADR no.	ADR manifestation (MedDRA® PT)	Type	Implicated drug(s)	Seriousness	Causality	Preventable
54	Male	Preterm neonate	0.9		Indeterminate	67	Renal impairment	A	Amphotericin + vancomycin	Increased monitoring, no harm	Probable	No
55	Male	Preterm neonate	0.8		Negative	68	Convulsion	A	Imipenem-cilastatin	Requires treatment, temporary harm	Probable	No
56	Female	Term neonate	3.1		Negative	69	Hypernatraemia	A	Sodium chloride	Increased monitoring, no harm	Definite	No
57	Female	Preterm neonate	1.9		Indeterminate	70	Convulsion	A	Morphine	Increased monitoring, no harm	Possible	No
						71	Hyponatraemia	A	Spironolactone + furosemide	Requires treatment, temporary harm	Possible	No
						72	Normochromic normocytic anaemia	A	Zidovudine	Requires treatment, temporary harm	Possible	Yes
58	Male	Infancy		-2.4	Indeterminate	73	Diarrhoea	A	Gentamicin + ampicillin	Increased monitoring, no harm	Possible	No
59	Female	Infancy		-4.4	Indeterminate	74	Hyperkalaemia	A	Potassium chloride	Increased monitoring, no harm	Definite	Yes
60	Male	Infancy		-2.0	Negative	75	Dermatitis diaper	A	Amoxicillin	Requires treatment, temporary harm	Possible	No
61	Female	Infancy		-5.2	Negative	76	Diarrhoea	A	Ampicillin	Requires treatment, temporary harm	Possible	No
						77	Renal failure acute	A	Furosemide + gentamicin + spironolactone	Requires treatment, temporary harm	Probable	Yes
62	Male	Infancy		-1.9	Negative	78	Hypokalaemia	A	Furosemide	Requires treatment, temporary harm	Possible	No
						79	Renal impairment	A	Gentamicin	Requires treatment, temporary harm	Probable	Yes
21	Female	Infancy		0.5	Indeterminate	80	Normochromic normocytic anaemia	B	Co-trimoxazole	Increased monitoring, no harm	Possible	No
63	Female	Infancy		-0.5	Negative	81	Diarrhoea	A	Ampicillin + clarithromycin + gentamicin	Increased monitoring, no harm	Possible	No
64	Male	Infancy		0.0	Negative	82	Diarrhoea	A	Amoxicillin + gentamicin + ampicillin	Requires treatment, temporary harm	Possible	No
65	Female	Infancy		-1.8	Positive	83	Hypokalaemia	A	Fenoterol	Increased monitoring, no harm	Possible	No
						84	Hyponatraemia	A	Co-trimoxazole	Increased monitoring, no harm	Possible	No
						85	Injection site phlebitis	A	Ganciclovir	Requires treatment, temporary harm	Possible	No
						86	Microcytic anaemia	A	Ganciclovir + co-trimoxazole	Requires treatment, temporary harm	Possible	No
						87	Oral candidiasis	A	Gentamicin + prednisone + ertapenem + ampicillin + azithromycin	Requires treatment, temporary harm	Possible	No
66	Male	Infancy		-1.9	Negative	88	Rash erythematous	B	Midazolam	Requires treatment, temporary harm	Possible	No
									Fluconazole		Possible	No
									Ganciclovir		Possible	No
									Co-trimoxazole		Possible	No
									Diazepam		Possible	No
									Clonidine		Possible	No
									Gentamicin		Possible	No

								Ampicillin		Possible	No
								Morphine		Possible	No
67	Male	Infancy	-2.0	Negative	89	Diarrhoea	A	Gentamicin + ampicillin	Increased monitoring, no harm	Possible	No
68	Male	Infancy	-5.4	Indeterminate	90	Diarrhoea	A	Ampicillin + gentamicin + amoxicillin	Increased monitoring, no harm	Possible	No
69	Male	Infancy	-0.5	Negative	91	Hyperkalaemia	A	Potassium chloride	Increased monitoring, no harm	Definite	No
70	Female	Infancy	-2.7	Negative	92	Coagulopathy	A	Heparin	Requires treatment, temporary harm	Probable	Yes
					93	Hyperkalaemia	A	Potassium chloride	Increased monitoring, no harm	Probable	No
71	Male	Infancy	-6.4	Indeterminate	94	Thrombocytopenia	B	Heparin	Increased monitoring, no harm	Probable	No
72	Female	Infancy	-4.4	Positive	95	Hyponatraemia	A	Co-trimoxazole	Increased monitoring, no harm	Possible	No
73	Female	Infancy	-4.6	Negative	96	Hyperkalaemia	A	Potassium chloride	Increased monitoring, no harm	Probable	No
74	Male	Infancy	2.0	Negative	97	Diarrhoea	A	Gentamicin + ampicillin	Increased monitoring, no harm	Possible	No
75	Male	Infancy	2.0	Negative	98	Rash maculo-papular	B	Amoxicillin	Increased monitoring, no harm	Possible	No
76	Female	Infancy	-2.6	Negative	99	Hypokalaemia	A	Furosemide	Requires treatment, temporary harm	Possible	No
77	Female	Infancy	-1.8	Negative	100	Hyperkalaemia	A	Potassium chloride	Increased monitoring, no harm	Probable	No
78	Male	Infancy	-0.5	Negative	101	Diarrhoea	A	Gentamicin + ampicillin + oseltamivir	Increased monitoring, no harm	Possible	No
79	Female	Infancy	-4.3	Indeterminate	102	Gastroenteritis clostridial	A	Meropenem + ertapenem + cloxacillin	Increased monitoring, no harm	Probable	No
80	Male	Infancy	-2.8	Negative	103	Hypokalaemia	A	Furosemide	Requires treatment, temporary harm	Possible	No
81	Male	Infancy	-3.9	Negative	104	Diarrhoea	A	Co-amoxiclav	Increased monitoring, no harm	Possible	No
82	Male	Infancy	-2.1	Negative	105	Diarrhoea	A	Ceftriaxone	Increased monitoring, no harm	Possible	No
83	Male	Infancy	-6.0	Indeterminate	106*	Hypokalaemia	A	Furosemide	Increased monitoring, no harm	Possible	No
					107*	Hypokalaemia	A	Furosemide	Requires treatment, temporary harm	Possible	No
					108	Hyponatraemia	A	Furosemide + spironolactone	Requires treatment, temporary harm	Possible	No
84	Male	Infancy	-0.1	Negative	109	Hypokalaemia	A	Salbutamol + aminophylline	Requires treatment, temporary harm	Possible	No
					110	Tachycardia	A	Salbutamol + aminophylline	Increased monitoring, no harm	Probable	No
85	Male	Infancy	-2.0	Negative	111	Diarrhoea	A	Co-amoxiclav	Increased monitoring, no harm	Possible	No
86	Male	Infancy	-2.4	Positive	112	Hyperkalaemia	A	Potassium chloride	Requires treatment, temporary harm	Definite	No
4	Male	Infancy	-5.5	Indeterminate	113	Hyperkalaemia	A	Potassium chloride	Requires treatment, temporary harm	Definite	Yes
87	Female	Infancy	-1.1	Indeterminate	114	Diarrhoea	A	Gentamicin + amoxicillin + ampicillin	Increased monitoring, no harm	Possible	No
88	Male	Toddler	1.0	Indeterminate	115	Diarrhoea	A	Ceftriaxone + ampicillin	Increased monitoring, no harm	Possible	Yes
89	Male	Toddler	0.5	Negative	116	Diarrhoea	A	Clarithromycin + ceftriaxone	Increased monitoring, no harm	Possible	No
90	Male	Toddler	-2.2	Negative	117	Diarrhoea	A	Ampicillin + amoxicillin	Increased monitoring, no harm	Possible	No
91	Male	Toddler	-1.6	Negative	118	Diarrhoea	A	Co-amoxiclav	Increased monitoring, no harm	Probable	Yes
					119	Hyperkalaemia	A	Potassium chloride	Increased monitoring, no harm	Probable	Yes

92	Female	Toddler		1.6	Indeterminate	120	Diarrhoea	A	Amoxicillin + ampicillin + albendazole	Increased monitoring, no harm	Possible	No
93	Male	Toddler		-0.8	Negative	121	Hyponatraemia	A	Furosemide	Requires treatment, temporary harm	Possible	No
94	Female	Toddler		-1.2	Negative	122	Diarrhoea	A	Phenoxymethylpenicillin + amoxicillin	Increased monitoring, no harm	Possible	No
95	Male	Toddler		-0.1	Negative	123	Diarrhoea	A	Amoxicillin	Increased monitoring, no harm	Possible	No
96	Female	Early childhood		-3.4	Negative	124	Diarrhoea	A	Mebendazole + rifampicin + amoxicillin + ceftriaxone	Requires treatment, temporary harm	Possible	No
41	Male	Early childhood		0.0	Negative	125	Hyperkalaemia	A	Potassium chloride	Increased monitoring, no harm	Probable	Yes
97	Female	Early childhood		-1.0	Negative	126	Oral candidiasis	A	Co-amoxiclav + prednisone	Requires treatment, temporary harm	Probable	No
98	Male	Early childhood		1.7	Negative	127	Hypokalaemia	A	Hydrochlorothiazide	Requires treatment, temporary harm	Possible	No
99	Male	Early childhood		-3.9	Positive	128	Vomiting	A	Co-trimoxazole	Increased monitoring, no harm	Possible	No
100	Male	Early childhood		-0.6	Negative	129	Tachycardia	A	Salbutamol + fenoterol	Increased monitoring, no harm	Possible	No
101	Male	Early childhood		1.7	Negative	130	Diarrhoea	A	Gentamicin + amikacin + mineral salts in combination + lactulose + ampicillin + co-amoxiclav	Increased monitoring, no harm	Probable	No
102	Male	Early childhood		2.7	Negative	131	Vomiting	A	Nitrous oxide + sevoflurane + ketamine	Increased monitoring, no harm	Possible	No
103	Female	Early childhood		0.3	Negative	132	Hypokalaemia	A	Fenoterol	Requires treatment, temporary harm	Possible	No
						133	Tachycardia	A	Fenoterol	Increased monitoring, no harm	Possible	No
104	Female	Early childhood		-3.0	Negative	134	Hypokalaemia	A	Furosemide + salbutamol + prednisone	Increased monitoring, no harm	Probable	No
105	Female	Early childhood		-1.5	Negative	135	Hypokalaemia	A	Prednisolone + prednisone + methylprednisolone	Requires treatment, temporary harm	Probable	No
						136	Hypophosphataemia	A	Sucralfate + prednisolone + methylprednisolone + dexamethasone + prednisone	Requires treatment, temporary harm	Probable	No
						137	Renal impairment	A	Ibuprofen + gentamicin	Increased monitoring, no harm	Possible	No
						138*	Upper gastrointestinal haemorrhage	A	Ibuprofen	Requires treatment, temporary harm	Definite	No
						139*	Upper gastrointestinal haemorrhage	A	Ibuprofen + prednisone + prednisolone + methylprednisolone	Requires treatment, temporary harm	Definite	No
106	Female	Early childhood		1.2	Negative	140	Rash erythematous	B	Phenoxymethylpenicillin	Increased monitoring, no harm	Possible	No
									Omeprazole		Possible	No
									Furosemide		Possible	No
107	Male	Early childhood		-0.8	Negative	141	Convulsion	A	Ertapenem	Requires treatment, temporary harm	Possible	No
108	Male	Early childhood		-0.7	Negative	142	Tachycardia	A	Fenoterol	Increased monitoring, no harm	Possible	No
109	Male	Early childhood	22		Negative	143	Hypokalaemia	A	Salbutamol + fenoterol	Requires treatment, temporary harm	Probable	No

110	Female	Early childhood	28	Negative	144	Hypokalaemia	A	Salbutamol	Requires treatment, temporary harm	Probable	No
					145	Tachycardia	A	Salbutamol	Increased monitoring, no harm	Probable	No
32	Female	Early childhood	23	Negative	146	Diarrhoea	A	Ertapenem + meropenem	Increased monitoring, no harm	Possible	No
111	Male	Middle childhood	18	Negative	147	Tachycardia	A	Salbutamol + fenoterol	Increased monitoring, no harm	Possible	No
112	Female	Middle childhood	23	Negative	148	Tachycardia	A	Salbutamol + fenoterol	Increased monitoring, no harm	Probable	No
113	Female	Middle childhood	17	Negative	149	Hyponatraemia	A	Furosemide	Requires treatment, temporary harm	Probable	No
114	Male	Middle childhood	24	Negative	150	Tachycardia	A	Salbutamol + fenoterol	Increased monitoring, no harm	Definite	No
115	Male	Middle childhood	17	Negative	151	Hallucination	A	Methylphenidate	Increased monitoring, no harm	Probable	No
					152	Peritonitis	A	Peritoneal dialytics	Requires treatment, temporary harm	Definite	No
116	Female	Middle childhood	22	Negative	153	Renal impairment	Mixed	Furosemide	Requires treatment, temporary harm	Possible	No
								Captopril		Possible	No
								Ibuprofen		Possible	No
								Amikacin		Possible	No
35	Female	Middle childhood	38	Negative	154	Hyperkalaemia	A	Potassium chloride	Increased monitoring, no harm	Definite	No
117	Male	Middle childhood	23	Positive	155	Hyponatraemia	A	Co-trimoxazole	Increased monitoring, no harm	Possible	No
118	Male	Early adolescence	Missing	Negative	156	Constipation	A	Morphine	Requires treatment, temporary harm	Definite	No
119	Female	Early adolescence	28	Negative	157	Gastroenteritis clostridial	A	Ertapenem + cefotaxime	Requires treatment, temporary harm	Probable	No
					158	Hypertension	A	Prednisone + tacrolimus	Requires treatment, temporary harm	Possible	No
					159	Hypokalaemia	A	Furosemide	Requires treatment, temporary harm	Possible	No
19	Female	Early adolescence	42	Negative	160	Hypokalaemia	A	Polystyrene sulfonate	Requires treatment, temporary harm	Definite	Yes

MedDRA® PT: Medical Dictionary for Regulatory Activities preferred term; WFA-z: weight-for-age z-score

* Two distinct episodes of the same ADR occurred during the patient's admission

Supplementary Table S12. Drug classes implicated in non-serious adverse drug reactions at two children's hospitals, South Africa, 2015.

ATC code	Description	Implicated in *n* non-serious ADRs
J01	Systemic antibacterials	48
R03	Drugs for obstructive airway diseases	23
C03	Diuretics	17
A12	Mineral supplements	11
H02	Systemic corticosteroids	10
J05	Direct-acting antivirals	6
M01	Anti-inflammatories and antirheumatics	4
B01	Antithrombotics	3
N02	Analgesics	3
A02	Drugs for acid-related disorders	2
J02	Systemic antimycotics	2
N05	Psycholeptics	2
P02	Antihelmintics	2
V03	Other therapeutic products	2
A03	Drugs for functional gastrointestinal disorders	1
A06	Drugs for constipation	1
B05	Blood substitutes and perfusion solutions	1
C02	Antihypertensives	1
C09	Agents acting on renin-angiotensin system	1
J04	Antimycobacterials	1
L04	Immunosuppressants	1
N01	Anaesthetics	1
N03	Antiepileptics	1
N06	Psychoanaleptics	1
R06	Systemic antihistamines	1
